# Cooperative Redox Catalysis of N_2_O Decomposition by Short‐Range RhO*_x_* and CeO*_x_* Anchored to Co_3_O_4_

**DOI:** 10.1002/anie.7890563

**Published:** 2026-06-23

**Authors:** Daniel C. Cano‐Blanco, Silvio Bellomi, Daniele Bonavia, Ivo Alxneit, Kirill A. Lomachenko, Karin Föttinger, Oliver Kröcher, Davide Ferri

**Affiliations:** ^1^ PSI Center for Energy and Environmental Sciences Paul Scherrer Institute Villigen PSI Switzerland; ^2^ Institute of Chemical Sciences and Engineering École polytechnique fédérale de Lausanne (EPFL) Lausanne Switzerland; ^3^ Institute of Materials Chemistry Technische Universität Wien Vienna Austria; ^4^ European Synchrotron Radiation Facility Grenoble France

**Keywords:** cooperative redox mechanism, modulated excitation XAS, N_2_O decomposition, spinel oxides

## Abstract

Hybrid materials based upon mixed metal oxides provide an effective strategy to boost catalytic performance through redox‐induced interfacial engineering. Herein, we present an efficient catalyst toward N_2_O decomposition containing short‐range ordered RhO*_x_* and CeO*_x_* species anchored to the Co_3_O_4_ spinel. The activity enhancement relative to Co_3_O_4_ is non‐additive with respect to the RhO*_x_*/Co_3_O_4_ and CeO*_x_*/Co_3_O_4_ interfaces and is uniquely linked to the modulation of the Co and Rh oxidation states at a synergistic Rh–O–Co–O–Ce interface. This ensemble provides increased redox flexibility and facilitated lattice oxygen activation, resulting in markedly enhanced N_2_O decomposition. Kinetic analysis and *operando* modulated excitation x‐ray absorption spectroscopy (ME‐XAS) provide direct mechanistic evidence that N_2_O decomposition proceeds via a multi‐site, cooperative Mars‐van Krevelen pathway, in which Co_3_O_4_–CeO_2_ acts as an oxygen reservoir enabling the redox cycling of RhO*_x_* species. This study highlights the ability of mixed metal oxide catalysts to exploit interfacial effects to achieve improved activity in redox‐mediated reactions and offers a rationale for the engineering of metal oxide–metal oxide catalysts guided by *operando* spectroscopy.

## Graphical abstract

A short‐range Rh–O–Co–O–Ce interfacial moiety drives low‐temperature N_2_O decomposition via a cooperative redox cycle. *Operando* modulated‐excitation XAS with phase‐sensitive detection (ME‐XAS + PSD) tracks rapid, reversible Rh oxidation coupled to Co reduction. Combined kinetics and DFT rationalize the activity trends and identify the Rh–O–Co interface as a key descriptor.

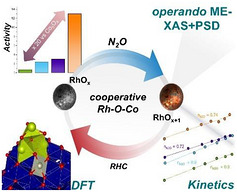

## 1 Introduction

Noble metals (NM) are scarce and costly, yet they present unique catalytic properties across a wide range of catalytically driven chemical transformations [1, 2]. To improve their utilization, large research efforts have been devoted to the design of catalysts, evolving from nanoparticles to short‐range ordered structures and, ultimately, to single‐atom catalysts [3]. While these approaches improve atom efficiency and often enhance activity, they also pose challenges in terms of stability, electronic control, and unambiguous identification of active sites [4].

In this context, metal oxide‐metal oxide hybrid materials have emerged as promising support materials for noble metals to address these limitations, because these systems often display markedly interfacial metal–support effects [5, 6, 7]. These interactions can effectively impact catalytic performance [8, 9, 10, 11], while simultaneously providing a platform for catalysts engineering, as they can stabilize noble metal atoms anchored at the oxide interface [12, 13]. These effects have been extensively rationalized in terms of tuning of the metal d‐band centers [5, 14].

Cobalt oxide (Co_3_O_4_) is a particularly attractive metal oxide support owing to its mixed‐valence (Co^2+^, Co^3+^) spinel structure, intrinsic redox activity, and tunable d‐orbital occupancy [15, 16]. Noble metals (Pt [11, 12, 17, 18], Pd [12], Ir [19], Ag [20], Au [21, 22], Rh [23, 24]) supported on Co_3_O_4_ have drawn increasing attention due to the tunable NM/Co_3_O_4_ interface, and have shown promise in reactions such as CO oxidation [11, 17, 21, 22], H_3_NBH_3_ hydrolysis [18], and VOC oxidation reactions (e.g., toluene, benzene, etc.) [25]. Moreover, short‐range‐ordered NM species anchored on Co_3_O_4_ are highly effective motifs for oxygen evolution reactions [26, 27, 28]. Beyond interfacial effects, bulk substitution strategies (Co_3_O_4_‐X, X = Zn, Al, Mg, Mn, Ni, Ce, Cr, etc.) can further tailor the electronic and redox properties of Co_3_O_4_ [29, 30, 31, 32, 33].

Despite advances in the design of Co_3_O_4_‐based catalysts, disentangling the roles of individual elements and precisely identifying the redox‐active sites under realistic conditions have proven difficult, as subtle changes are often masked by the bulk Co_3_O_4_ response [11, 16, 24, 28, 34]. *Operando* transient methods, particularly modulated excitation (ME) with phase‐sensitive detection (PSD), have therefore emerged as powerful tool for gaining molecular‐level insights into the structural and electronic dynamics of complex catalytic systems that are otherwise inaccessible using conventional methods [35, 36].

Direct N_2_O decomposition into harmless N_2_ and O_2_ serves as an ideal probe reaction to interrogate interfacial properties in Co_3_O_4_‐X‐supported noble metal catalysts. Its mechanism is governed by structural, electronic, and redox properties of active sites that can be modulated at the metal–Co_3_O_4_ interface [37, 38]. Beyond the mechanistic relevance, its chemistry has remarkable environmental importance: N_2_O is a potent greenhouse gas with a global warming potential ca. 310 times greater than CO_2_ [39], and its emission has been identified as a key challenge in the future of carbon neutral transportation and energy sectors [40, 41]. Notably, the introduction of N_2_O among regulated pollutants in the Euro VII emissions standards [42] and its potential formation in next‐generation combustion engines [40, 43, 44], highlights both its environmental significance and the technical challenges of its abatement. Hence, the development of effective technologies for N_2_O mitigation has become crucial [44, 45].

In this work, we designed a mixed‐oxide RhO*_x_*/Co_3_O_4_‐CeO_2_ catalyst, exploiting the complementary role of its components. Rh provides highly active sites for N─O bond activation [46, 47], while Co_3_O_4_‐CeO_2_ offers a redox‐active structure with tunable properties of the Rh/oxide interface [46, 48, 49, 50, 51]. To disentangle these contributions, we systematically investigated Co_3_O_4_, Co_3_O_4_‐CeO_2_, RhO*_x_*/Co_3_O_4_, and RhO*_x_*/Co_3_O_4_‐CeO_2_ catalysts using complementary *ex situ* characterization, DFT modeling, kinetic analyses, and N_2_O decomposition as the probe reaction. By coupling *operando* x‐ray absorption spectroscopy (XAS)—mass spectrometry (MS) with ME‐PSD, we identify a cooperative reaction mechanism between RhO*_x_* sites and Co ions sites on the most active catalyst in the series, RhO*_x_*/Co_3_O_4_‐CeO_2_.

## 2 Results and Discussions

### 2.1 Structural Characterization and Local Geometry

**FIGURE 1:**
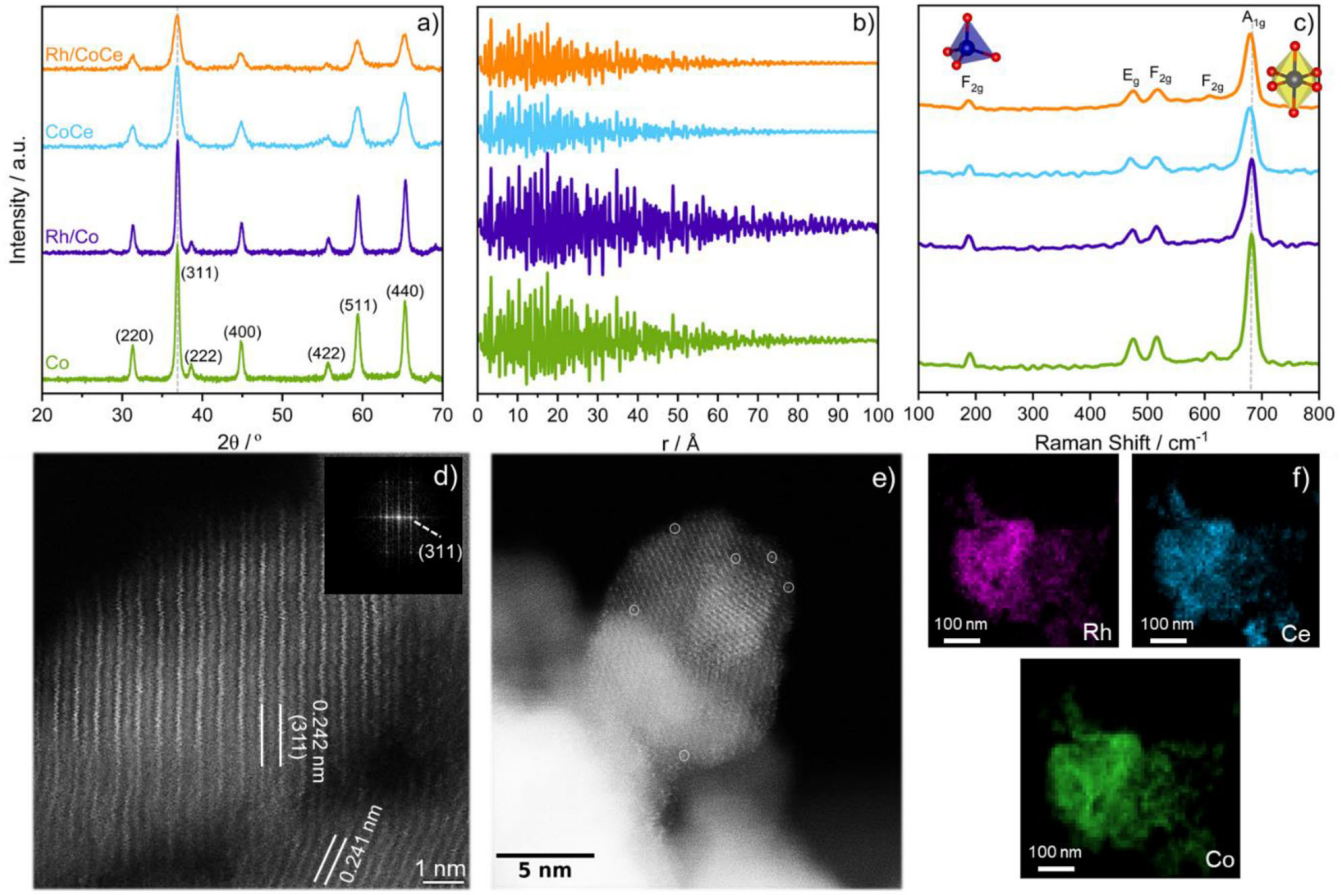
(a) PXRD patterns, (b) pair distribution function data (PDF), and (c) Raman spectra of Co, CoCe, Rh/Co, and Rh/CoCe. (d and e) HAADF‐STEM images of Rh/CoCe_,_ and (f) EDX maps for Rh, Ce, and Co. The white circles in (e) highlight regions of higher contrast.

All catalysts exhibit the crystal structure of the cubic Co_3_O_4_ spinel (PDF#74‐1656; *Fd*‐3*m* space group symmetry; labeled as Co in Figure 1a), and display type‐IV adsorption–desorption isotherms (Figure S3). BET surface areas range from 64.9 m^2^ g^−1^ to 133.2 m^2^ g^−1^ (Table S1), increasing upon Ce incorporation (Co_3_O_4_‐CeO_2_, labeled as CoCe) and decreasing after Rh impregnation (Rh/Co_3_O_4_, Rh/Co; Rh/Co_3_O_4_‐CeO_2_, Rh/CoCe). No distinct reflections attributable to Rh‐containing phases are observed as indication of the high dispersion of Rh. Also, no CeO_2_ phase is detected, but the characteristic peaks of Co_3_O_4_ broaden due to the decrease in crystallite size and increase in density of defect sites upon addition of Ce [52, 53]. Along this line, experimental pair distribution function (PDF; Figure 1b) confirms that the short‐atomic correlations correspond to the crystal structure of Co_3_O_4_ [54] (Figure S4) and provides direct evidence that Ce addition decreases the coherent domain size in CoCe and Rh/CoCe_,_ due to the presence of a more defective, distorted lattice [55, 56]. Rh and Ce also have no significant effect on the Raman spectra (Figure 1c), which display the characteristic phonon modes of the Co_3_O_4_ spinel [57], dominated by the vibration of Co^3+^‐O units in octahedral coordination (Co_Oh_; A_1g_, 690 cm^−1^). Only this A_1g_ band redshifts and loses symmetry in Ce‐containing samples, consistent with the structural distortions in the Co_3_O_4_ lattice supported by powder x‐ray diffraction (PXRD) and PDF analysis. The observed changes reflect a modification of the local bonding environment of Co_Oh_ sites induced by CeO*_x_*, resulting in more strained and oxidized Co sites [50]. Incorporation of Ce into the Co_3_O_4_ or formation of a mixed solution is unlikely due to the marked difference in ionic radii between Co^3+^ (55–61 pm) and Ce^4+^ (101–114 pm) [50, 58].

High‐angle annular dark‐field scanning transmission electron microscopy (HAADF‐STEM) images of Rh/CoCe (Figure 1d) show that the material is composed of well‐crystallized Co_3_O_4_ host‐domains. The lattice spacing of ca. 0.241 nm in the HAADF‐STEM images and the corresponding inverse fast Fourier‐transform (IFFT) is assigned to the (311) planes of Co_3_O_4_. Instead of distinct RhO*_x_* or CeO_2_ nanoparticles, the isolated bright spots (high Z contrast) that are often aligned along the (311) lattice planes (Figure 1e) correspond to individual Rh or Ce atoms or very small clusters anchored to the surface of the spinel. Energy‐dispersive x‐ray (EDX) elemental maps (Figures 1f and S5) further confirm the high dispersion of Rh and Ce throughout the support. Together, the data confirms high dispersion and the absence of crystalline RhO*_x_* or CeO*_x_* domains. The short‐range nature of RhO*_x_* and Rh^+^ species anchored on Co_3_O_4_ is further corroborated by the infrared spectra of adsorbed CO (Figure S6).

**FIGURE 2:**
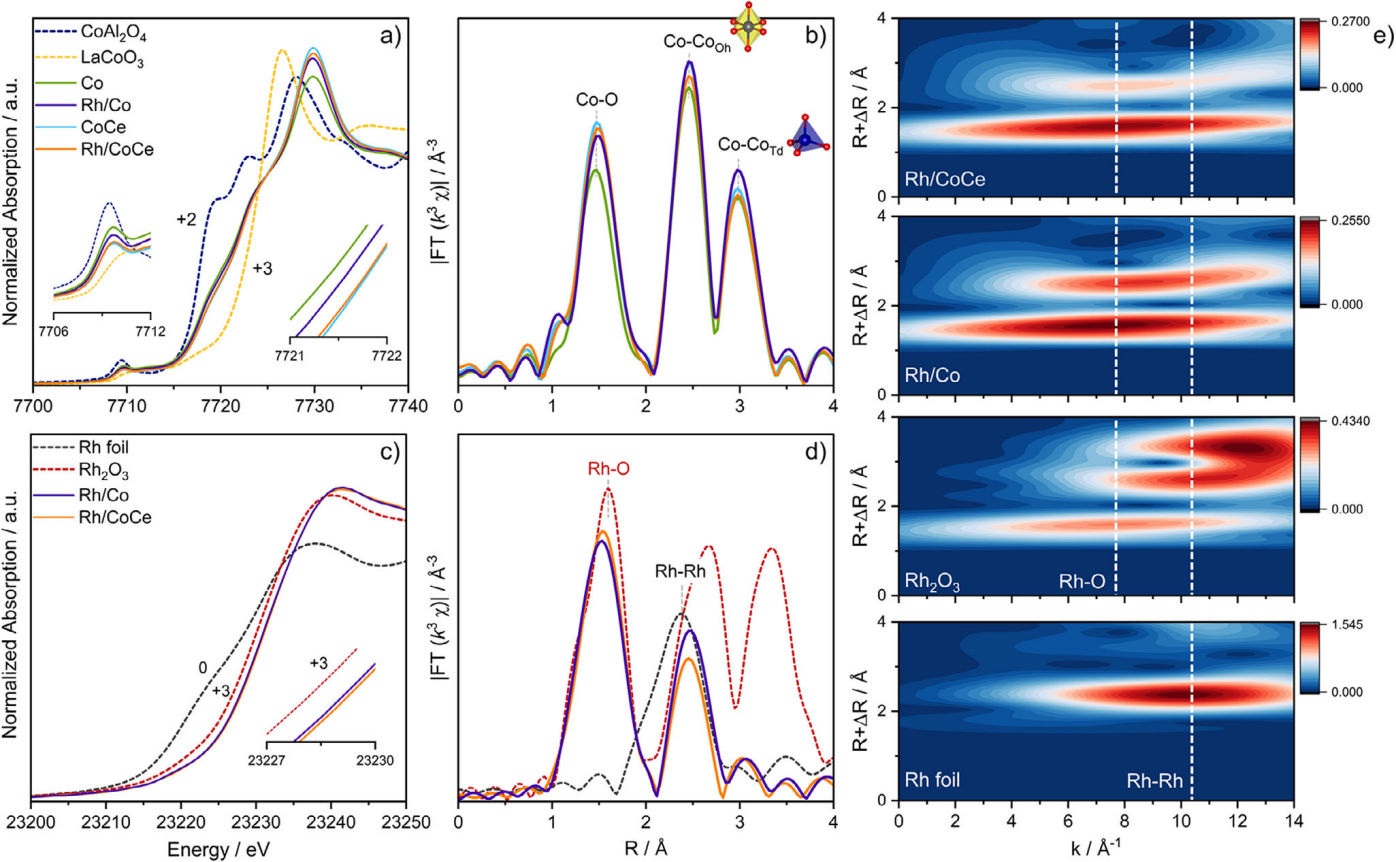
(a) Normalized Co K‐edge XANES spectra and (b) corresponding phase‐uncorrected *k* ^3^‐weighted magnitude of the FT‐EXAFS spectra of Co, CoCe, Rh/Co, Rh/CoCe, and reference samples (LaCoO_3_ and CoAl_2_O_4_). (c) Normalized Rh K‐edge XANES and (d) *k* ^3^‐weighted magnitude of FT‐EXAFS spectra of Rh/Co, Rh/CoCe, and reference spectra (Rh foil, scaled five times (/5), and Rh_2_O_3_), and (e) their corresponding *k* ^3^‐weighted wavelet‐transforms (WT).

Qualitative analysis of the edge position and pre‐edge features of Co K‐edge x‐ray absorption near edge spectra (XANES, Figure 2a) measured at ambient conditions indicates a mixed‐valence state of Co between +2 and +3. Both Ce and Rh shift the average valence state of Co toward a more oxidized character compared to Co_3_O_4_, in the order Rh/CoCe ∼ CoCe > Rh/Co > Co, that is confirmed by the increased white‐line intensity. The intensity of the pre‐edge feature, which characterizes symmetry effects in the 1s→3d transition and is sensitive to local coordination symmetry, also decreases for the Ce‐ and Rh‐containing samples relative to Co_3_O_4_, suggesting increased centrosymmetry and reduced 3d–4p mixing in the local Co coordination environment [59]. Three main coordination shells in the Co K‐edge Fourier‐transformed (FT) extended x‐ray absorption fine structure (EXAFS) spectra of all samples (Figure 2b) are assigned to Co─O bonds (*R* = 1.55 Å), Co_Oh_–Co_Oh_ pairs (2.57 Å), and Co_Td_–Co_Td_ pairs (3.1 Å), consistent with a mixed‐valence Co_3_O_4_ spinel structure [32, 50]. Rh and especially Ce cause a slightly higher Co–O coordination number (*N* = 5.4–6) compared to Co_3_O_4_ (5.2; Figures S7 and S8; Table S2), indicating a more fully developed octahedral Co environment, in line with the pre‐edge observations. This is consistent with a higher average Co oxidation state and stronger Co–O covalency, likely due to Co charge re‐distribution following electron transfer from Co_3_O_4_ to Ce [50, 60]. Therefore, while the average structure of the spinel lattice is preserved, Ce and Rh induce local structural distortion in the Co_3_O_4_ lattice [50], in agreement with the above structural characterization (Figure 1). The similar Rh‐K edge XANES spectra of Rh/Co and Rh/CoCe (Figure 2c) display an absorption edge at higher energies than that of Rh_2_O_3_, revealing an average oxidation state higher than +3 [47, 61]. Furthermore, the higher oxidation state in Rh/CoCe than in Rh/Co indicates that Ce can act as an electron reservoir facilitating charge transfer between Rh and Co sites [62], leading to a higher oxidation state of Rh and Co. The corresponding Rh K‐edge FT‐EXAFS spectra (Figure 2d) are dominated by a first coordination shell of oxygen neighbors (phase‐uncorrected *R* = 1.91 Å). Both Rh‐impregnated samples show comparable Rh‐O shell intensities with coordination numbers of ca. 5.4 ± 0.4 (Figures S9 and S10; Table S3). A second coordination shell emerges at higher *R* values (phase‐uncorrected *R* = 2.4 Å) that is distinct from the Rh–Rh scattering of metallic Rh, suggesting the presence of Rh–Co and Rh–Ce interactions. Wavelet‐transform (WT)‐EXAFS analysis of Rh/Co and Rh/CoCe (Figure 2e) confirms this hypothesis and reveals two distinct intensity regions. The first corresponds to a Rh–O coordination shell with a local maximum at *k* = 7.74 Å^−1^ and *R* = 1.56 Å, aligning with Rh_2_O_3_ and pointing toward the oxidized state of Rh. A second domain at *k* = 8.51 Å^−1^ and *R* = 2.49 Å disagrees with the characteristic Rh–Rh scattering of metallic Rh (*k* = 10.37 Å^−1^ and *R* = 2.37 Å), suggesting a second‐shell contribution from backscattering by lighter neighboring atoms [19, 24, 59]. In the case of Rh/CoCe, the latter maximum shifts to *k* = 8.06 Å^−1^ and *R* = 2.49 Å, indicative of an enhanced contribution of Rh‐Co(Ce) interactions, likely arising from stronger interface interactions and a higher degree of Rh dispersion. Because Rh–O–Ce neighbors are expected at values of *R* = 3.64 Å and at higher *k*, the observed results are more consistent with a dominant Rh–O–Co contribution [63, 64]. Notably, EXAFS fitting (Figures S9 and S10; Table S3) is well described by explicit Rh–Co and Rh–O–Co scattering paths, further supporting short‐range RhO*_x_* species rather than pure Rh–Rh contributions. The effect of Ce is reflected in a slightly longer Rh–Co distance (2.906 Å for Rh/Co vs. 2.920 Å for Rh/CoCe), consistent with the presence of a Rh–O–Co–O–Ce interface.

### 2.2 Electronic Structure and Reducibility Properties

The decomposition of N_2_O is considered to proceed via a redox mechanism, in which the desorption of surface oxygen (O_2_) is critical. Therefore, catalyst reducibility and the nature of surface oxygen species are key parameters to rationalize the catalyst behavior.

**FIGURE 3:**
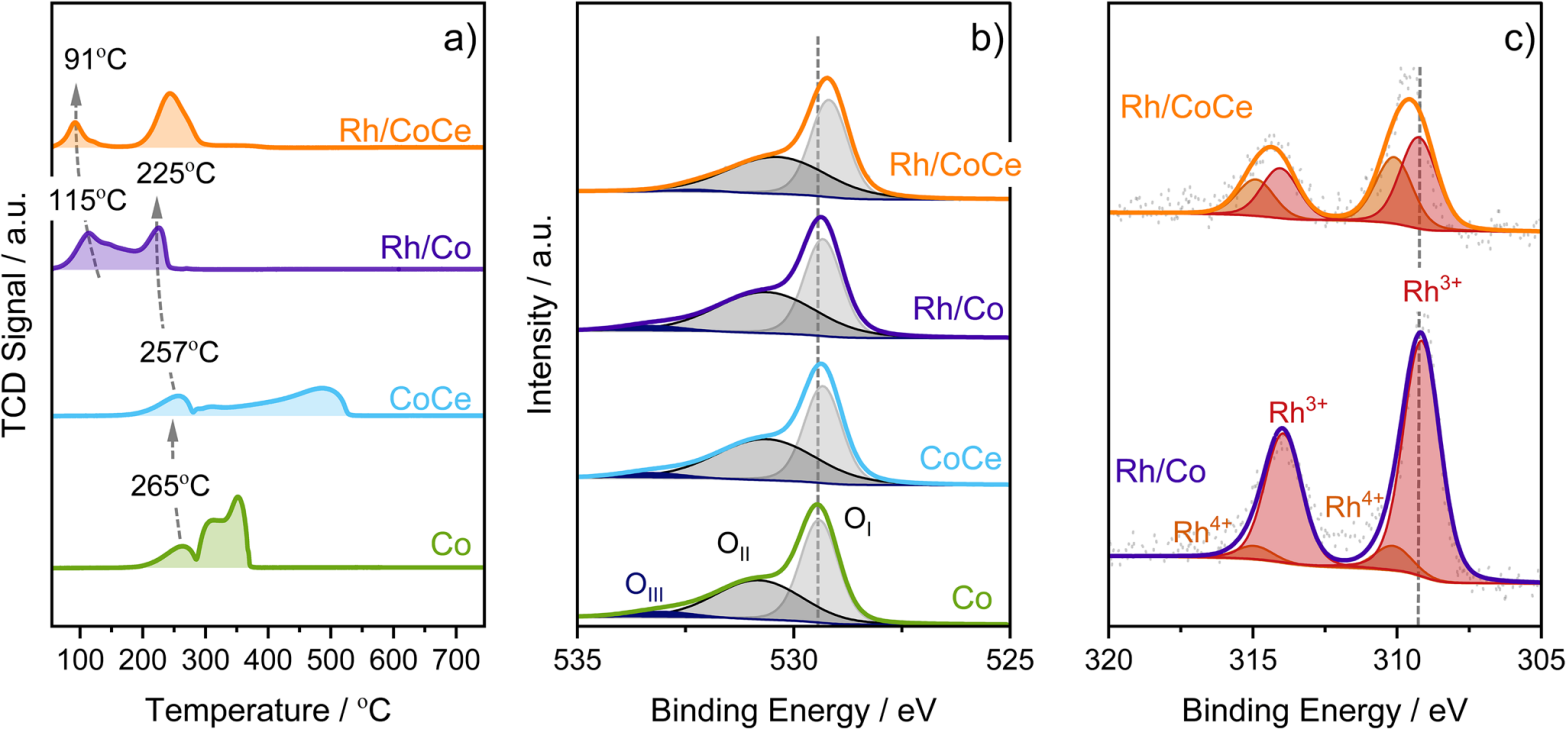
(a) H_2_ temperature programmed reduction (H_2_‐TPR) of Co, CoCe, Rh/Co, and Rh/CoCe. (b) O 1s XPS spectra of Co, CoCe, Rh/Co, and Rh/CoCe and (c) Rh 3d XPS spectra of Rh/Co and Rh/CoCe.

The two distinct reduction events in the H_2_ temperature‐programmed reduction (H_2_‐TPR) thermogram of Co_3_O_4_ (Figure 3a) characterize the reduction of Co^3+^ to Co^2+^ (Co_3_O_4_ → CoO, ca. 265°C) and the subsequent reduction of Co^2+^ to metallic Co (ca. 355°C) [65]. The shift of the former peak to lower temperature (Co_3_O_4_ → CoO, ca. 257°C) in CoCe [51, 65, 66] is associated with redox interactions between Co^3+^/Co^2+^ and Ce^4+^/Ce^3+^ redox couples [56, 66, 67], and is accompanied by a broadening of the high‐temperature reduction peak, suggesting decreased reducibility of Co^2+^ [65], but also a possible contribution from the reduction of Ce^4+^ that is facilitated by H_2_ spillover from Co. These findings are consistent with the Raman spectra [68, 69, 70] (Figure 1c), indicating that Ce primarily affects the Co^3+^–O environment (Co_Oh_ coordination) [71]. To confirm this, the incorporation of Rh in bulk Co_3_O_4_ was investigated by comparing the substitution of octahedral and tetrahedral sites (Figure S11). DFT simulations showed that the octahedral coordination of Rh is favored by 1.87 eV relative to the tetrahedral environment. This yields an oxidation state (+3.1) comparable to the XAS results, in contrast to that of the Rh in tetrahedral coordination (+2.7). Nonetheless, in line with previous studies [29, 32], the simulations also demonstrate that the incorporation of octahedral Rh induces a modification of the Co^2+^–O environment, specifically a slight contraction (average of 0.015 Å) of the surrounding tetrahedral Co^2+^–O bonds. However, this effect is expected to be limited, in agreement with the previous characterization results.

The Rh‐containing catalysts (Rh/Co and Rh/CoCe) exhibit an additional low‐temperature feature due to reduction of RhO*_x_* species [72, 73]. This peak appears at a lower temperature for Rh/CoCe than for Rh/Co, suggesting further improved reducibility due to the interaction between Rh, Co, and Ce. In both catalysts, the characteristic Co_3_O_4_ reduction peaks (Co^3+^ → Co^2+^ → Co^0^) collapse into a broader, single feature that shifts to lower temperature [74, 75]. This indicates enhanced oxygen mobility within the Co_3_O_4_ host lattice, as well as a promotional effect of Rh on the redox properties of Co (and Ce) species, likely due to Rh–Co(Ce) interactions [76, 77]. H_2_ spillover from Rh to Co_3_O_4_ may also promote the migration of bulk oxygen to the surface [78].

The low temperature region of the O_2_ temperature‐programmed desorption profiles (<400°C, O_2_‐TPD, Figure S12) corresponds to the desorption of surface oxygen species (O_ads_, i.e. O^2−^ and O^−^), while high‐temperature desorption (>400–700°C) is associated with the release of lattice oxygen (O_latt_) [79, 80]. Focusing on O_ads_, the incorporation of both Ce and especially Rh lowers the onset temperature for oxygen desorption. This implies a readier supply of active oxygen species at the surface, which is favorable for N_2_O decomposition, as it reflects the ability to recombine and remove O atoms formed during reaction [46]. Consistently, the oxygen‐desorption capacity obtained from integration of the thermograms follows the same order as the reducibility observed in the H_2_‐TPR profiles: Rh/CoCe > Rh/Co > CoCe > Co.

The oxidation state and oxygen lability were probed by x‐ray photoelectron spectroscopy (XPS, Figure 3b,c). The Co 2p spectra (Figure S13 and Table S4) display the characteristic spinel doublet consisting of components assigned to Co^3+^ (ca. 779.2 eV) and Co^2+^ (ca. 780.8–781.9 eV) and their satellites at ca. 10 and 6 eV from the main line, respectively [81]. The weak Co^2+^ satellite supports the predominant presence of a spinel Co_3_O_4_ phase [81, 82], consistent with the structural characterization. The surface Co^2+^/Co^3+^ ratio follows the order: Co (1.10) > Rh/Co (1.00) > CoCe (0.81) > Rh/CoCe (0.75), mirroring that obtained from Co K‐edge XANES, H_2_‐TPR, and O_2_‐TPD. This suggests that Rh and Ce stabilize Co^3+^ at the surface, enhancing oxygen mobility and the ability of the material to participate in Mars‐van Krevelen‐type reactions, involving lattice oxygen [33, 68, 83]. Three components were used to fit the O 1s spectra (Figure 3c and Table S5): lattice oxygen (O_I_, 529.5 eV), surface chemisorbed oxygen (O_II_, 530.5 eV), and hydroxyls (O_III_, 533.0 eV) [68, 83]. The O_I_ component of Co_3_O_4_ (529.41 eV) shifts to lower energy upon addition of Rh (529.31 eV in Rh/Co) and Ce (529.33 eV in CoCe), while Rh/CoCe possesses the lowest O_I_ value (529.19 eV). Overall, these changes indicate the formation of more reactive and covalent metal–oxygen bonds [66], corroborating the above characterization.

The influence of Ce on the surface redox behavior is evident. In the Ce 3d region of both CoCe and Rh/CoCe, the fit of the signals using a combination of Ce^3+^ and Ce^4+^ components reveals partially reduced CeO*_x_* (Figure S14 and Table S6) [84]. The spectra resemble those characteristic of ceria (CeO_2_), clearly differing from the signature of atomically dispersed Ce [85], in agreement with the observation from XRD and PDF that Ce does not incorporate in the spinel lattice, but forms well‐dispersed CeO*_x_* agglomerates visible by HAADF‐STEM but not large enough to be captured by XRD. In the presence of Rh, the Ce^3+^ fraction increases moderately from 0.36 (CoCe) to 0.40 (Rh/CoCe), pointing to an electronic coupling between Rh and the CeO_2_ agglomerates. The magnitude of this change argues against a structure in which Rh is hosted on these CeO_2_ domains [63]. Based on the structural characterization, the observed change is more consistent with a short‐range interaction between Rh and neighboring CeO_2_, known for its dynamic redox behavior and ability to act as electron reservoir [86], with both phases mutually influencing their respective redox properties. The Rh 3d spectra can be fit with only Rh^3+^ (ca. 309 eV) and Rh^4+^ (ca. 310 eV) components [87, 88], with Rh^3+^/Rh^4+^ ratios (Rh/Co, 9.59; Rh/CoCe, 1.37) demonstrating that Ce increases the Rh^4+^ content (Table S4) and the electronic coupling between Rh and CeO_2_.

**FIGURE 4:**
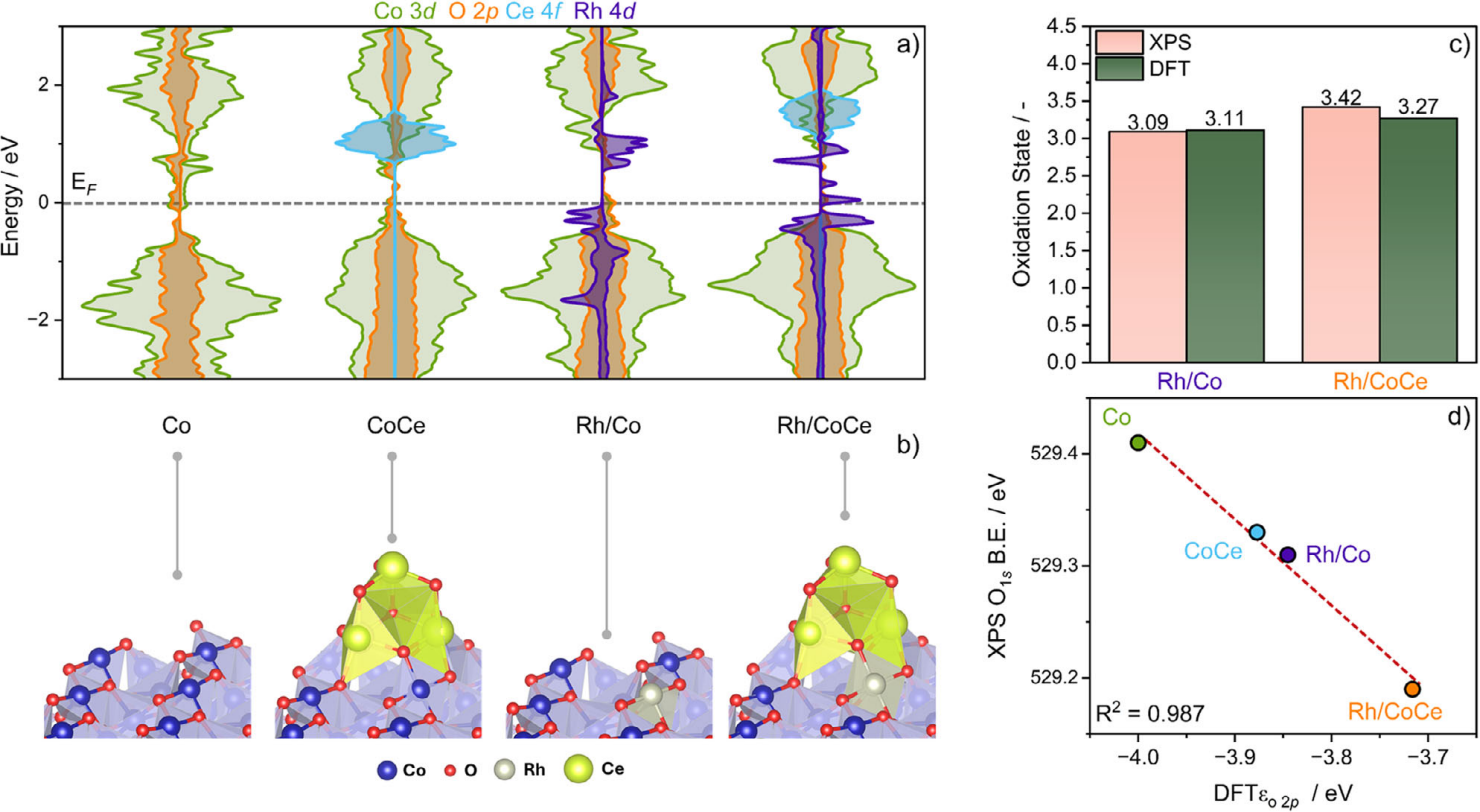
Electronic structure analysis. (a) Projected density of states (PDOS) and (b) structural models of Co, CoCe, Rh/Co, and Rh/CoCe. (c) Relation between the oxidation state of Rh from the Bader charges (DFT) and XPS data of Figure 3. (d) Correlation between the centroid of the O 2p band (*ε*_O2p_) from DFT and the lattice oxygen signal from the O 1s XPS narrow scans. The atoms in the DFT structures are identified in the legend.

DFT calculations were performed to gain atomic‐level insights into the experimentally observed redox behavior and to develop a rationale for the progressive reorganization of the electronic structure of Co_3_O_4_ upon addition of Ce and Rh, which ultimately impacts the surface and catalytic properties. Based on experimental characterization, CeO*_x_* agglomerates on the Co_3_O_4_ surface were modeled as Ce_4_O_7_ clusters, while oxidized Rh was represented as atomically dispersed on the spinel host. The relative position of RhO*_x_* and CeO*_x_* was determined by systematically screening different configurations (Figure S1), with the most stable arrangement forming Rh─O─Co─O─Ce bonds. This configuration confirms the hypothesis of short‐range interfacial interactions between RhO*_x_* and CeO*_x_*, providing a structural basis to interpret the electronic coupling observed experimentally. Furthermore, the excellent agreement between the fitted EXAFS results (Figures S7–S10) and the calculated scattering paths from the DFT structures indicates that the proposed models are consistent with the local structures proposed based on the experimental data. The projected density of states (PDOS) of the surfaces was analyzed, focusing on the band centers of the different elements (O 2p, Co 3d, Rh 4d, and Ce 4f), which are considered descriptors of the oxygen lability and redox flexibility in metal oxides [89, 90]. The PDOS data are shown in Figure 4a, and the corresponding structural models in Figure 4b.

The electronic structure of Co_3_O_4_ (Figure 4a) is dominated by Co─O─Co bonds. In a pristine spinel framework, the O 2p band lies well below the Fermi level (E_F_) (*ε*_2p_ = −4.03 eV), and the Co 3d states split into a bonding (*ε*_3d_ = −3.21 eV) and an upper band (*ε*_3d*_ = +2.02 eV). This electronic configuration matches a partially covalent Co–O interaction and a moderate lattice oxygen mobility [89, 90], mirroring the structural characterization. In CoCe, interfacial Co─O─Ce bonds form with localized unoccupied Ce 4f manifolds (*ε*_4f*_ = +1.09 eV) located between the occupied O 2p (*ε*_2p_ = −3.87 eV) and the unoccupied Co 3d (*ε*_3d*_ = +2.01 eV) states. These Ce 4f states act as electron buffer, providing intermediate electron‐accepting reservoirs that reduce the effective metal‐oxygen gap [90]. The result is enhanced activation of metal─oxygen bonds, supported by the XPS and H_2_‐TPR/O_2_‐TPD data.

When RhO*_x_* anchors to the surface of Co_3_O_4_, Rh–O–Co bridges are formed, leading to the reorganization of the electronic structure around the Rh states. In proximity of *E*_F_, a high density of occupied Rh 4d states is observed compared to Co 3d (*ε*_3d_ = −3.29 eV), implying that less energy is required for RhO*_x_* to participate in electron transfer [68]. The O 2p band shifts upwards (*ε*_2p_ = −3.71 eV), and the low‐lying Rh 4d antibonding states (*ε*_4d*_ = +1.15 eV) effectively become the lowest unoccupied levels (Co 3d *ε*_3d*_ = +2.20 eV). This decreases the effective metal–oxygen gap and improves the redox flexibility and mobility of oxygen [89, 90].

The most substantial restructuring of the electronic structure occurs when RhO*_x_* and CeO*_x_* coexist, and Rh–O–Co–O–Ce motifs are formed. The density of occupied Rh 4d states close to *E*_F_ increases with respect to Rh/Co, and the O 2p (*ε*_2p_ = −3.71 eV) and Co 3d (*ε*_3d_ = −3.02 eV) levels move upwards. The onset of the antibonding Rh 4d states located right above E_F_ lowers the upper‐band centroid (ε_4d*_ = +1.05 eV) and decreases the effective metal‐oxygen gap further. The Ce 4f states appear at higher energy (ε_4f_ _*_ = +1.52 eV), followed by the Co 3d (ε_3d_ _*_ = +2.26 eV). The proximity of the Ce 4f and Rh 4d states, mediated by bridging oxygen atoms, effectively couples Ce to Rh, in agreement with the increased content of Ce^3+^ sites observed from XPS. In a similar manner, the upward displacement of the Ce 4f* level toward the Co 3d* states strengthens the Ce–Co antibonding overlap, with the interconnecting oxygen atoms enabling efficient redox communication. Altogether, Rh 4d unoccupied states are now the lowest‐energy electron reservoir available for metal‐oxygen transfer, and Ce acts as a mediator of short‐range hybridization between Co and Rh [90]. This produces a single, integrated network in which electron redistribution between Rh, Ce and Co is energetically favored, stabilizing Rh in a higher, more reactive, oxidation state. The increase of the average Rh oxidation state from +3.09 (Rh/Co) to +3.42 (Rh/CoCe) observed in XPS is consistent with the values obtained from the DFT Bader charges of +3.11 (Rh/Co) and +3.27 (Rh/CoCe) (Figures 4c and S15).

The different bonding patterns and the resulting electronic coupling greatly impact the activation of metal–oxygen bonds and the reactivity of oxygen, which is experimentally probed by O_2_‐TPD and H_2_‐TPR. The O 2p states, representing the valence electrons most involved in metal‐oxygen bonding, shift upward from Co (−4.04 eV) to Rh/CoCe (−3.72 eV), indicating more reactive and delocalized electrons, correlating with a smaller energy gap with the metal bands and stronger hybridization [86]. These more delocalized and energetic electrons can screen the photoelectric core‐hole generated in the O 1s orbital more effectively, lowering the resulting binding energy [91], as reflected by the linear correlation observed between the DFT *ε*_2p_ and the XPS binding energies of the O 1s main photoemission line (Figure 4d). DFT thus demonstrates that Rh and Ce generate a synergistic mixed framework in which bond‐specific interactions induce cooperative electronic effects. The progression from Co–O to Co–O–Ce and to Co–O–Rh–O–Ce represents a systematic expansion of the metal–oxygen connectivity, defining the enhanced redox flexibility and lattice oxygen activation of Rh/CoCe.

### 2.3 N_2_O Decomposition and Reaction Kinetics

**FIGURE 5:**
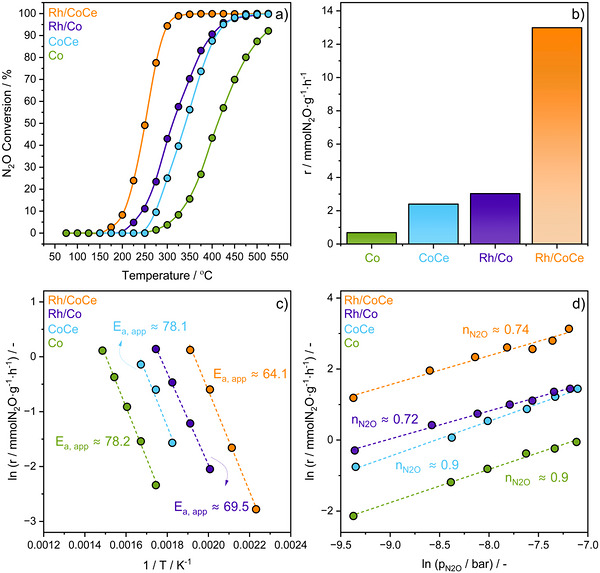
Catalytic behavior of Co, CoCe, Rh/Co, and Rh/CoCe. (a) N_2_O conversion as a function of reaction temperature. (b) Reaction rates measured under differential reactor conditions at 375°C. (c) Arrhenius plots derived from N_2_O light‐off curves and (d) effect of N_2_O partial pressure on reaction rates. Unless otherwise stated, all experiments were conducted with 500 ppmv N_2_O, 2 vol% O_2_, N_2_ balance, at 1 atm, total flow rate of 100 mL min^−1^, and variable catalyst mass (0–50 mg). *E*_a,app_ is expressed in kJ mol^−1^.

We next investigate the effects of the formation of the Rh–O–Co–(O–Ce) interface on Co_3_O_4_ and establish structure–activity relationships for the N_2_O decomposition (Figure 5a). Co_3_O_4_ exhibits moderate activity, with N_2_O conversion commencing at ca. 275°C and a light‐off temperature (*T*_50_) of ca. 412°C. CoCe shows significantly earlier onset temperature at ca. 250°C, and a value of *T*_50_ of ca. 339°C, indicating improved redox behavior due to the increased oxygen mobility. Rh/Co further decreases the onset temperature (ca. 200°C) with a *T*_50_ value of ca. 312°C, and the highest activity is observed for Rh/CoCe, which reaches complete N_2_O conversion in the 325°C–525°C temperature window, with an onset temperature of ca. 175°C and a *T*_50_ value of ca. 250°C. The order of reactivity suggested by the light‐off curves is corroborated by reaction rate values measured under differential reaction conditions (χ_N2O_ < 30%; Figure 5b and Table S7), allowing comparison among the samples in the absence of mass transport limitations. Relative to the Co_3_O_4_ spinel (0.67 mmol N_2_O g_cat_^−1^ h^−1^), CoCe exhibits a 3.5‐fold activity enhancement (2.38 mmol N_2_O g_cat_^−1^ h^−1^), Rh impregnation delivers a 4.3‐fold increase (3.01 mmol N_2_O g_cat_^−1^ h^−1^), and Rh/CoCe shows a ca. 20‐fold increase (12.96 mmol N_2_O g_cat_^−1^ h^−1^). These values align with, and in some cases surpass, previous data (Table S8), underscoring the critical role of Rh and CeO_2_ in promoting N_2_O decomposition. The pronounced variations in activity correlate with the differences in redox and structural properties among the catalysts, including enhanced lattice oxygen activation and covalency.

This does not seem to be able to counteract the typical inhibitory effect of water and oxygen (Figure S16). H_2_O (10 vol%) suppresses Rh/CoCe activity, shifting *T*_50_ from 250°C to 430°C (Figure S16a), while O_2_ (10 vol%) lowers N_2_O conversion by ca. 33% at 250°C (Figure S16b). Consistent with the O_2_ inhibitory effect, Rh/CoCe loses also ca. 30% of N_2_O conversion after 78 h on stream (Figure S17), likely due to accumulation of oxygen containing surface species [90, 92].

Arrhenius plots (Figure 5c) show consistently higher rates for Rh/CoCe across the examined temperature range. The apparent pre‐exponential factor (ln *A*_pp;_ Table S7) increases from 14.1 (Co_3_O_4_) to 15.6 (CoCe), consistent with structural differences influencing the effective collision frequency and/or exposed site density for N_2_O at active Co sites. In contrast, the similar ln *A*_pp_ values for Rh/Co and Rh/CoCe (14.9) suggest a comparable Rh speciation/ensemble. Apparent activation energies (*E*_a,app_) for the Co_3_O_4_ spinel (78.2 kJ mol^−1^) align with reported values (Table S9) [65, 68, 93]. CoCe and Co_3_O_4_ show comparable *E*_a,app_ values (ca. 78 kJ mol^−1^), suggesting that the same family of active sites is involved in N_2_O decomposition over these materials and that the rate‐determining step (RDS) is not affected by CeO_2_. In contrast, Rh in Rh/Co induces a significant decrease of *E*_a,app_ to ca. 69.5 kJ mol^−1^; we associate this change with a modification in the nature of the active sites and the mechanistic pathway. This value decreases further to 64.1 kJ mol^−1^ for Rh/CoCe, pointing toward a more efficient reaction pathway following the interaction between Rh and CeO_2_.

Because N_2_O decomposition operates at elevated temperatures, entropic effects are non‐negligible [48, 94]. Thus, we complemented the Arrhenius analysis with transition‐state theory using Eyring plots (Figure S18). For all catalysts, the enthalpy of reaction (∆*H* ^‡^) values agree with the apparent Arrhenius *E*_a,app_ in the studied temperature range, consistent with a single dominant RDS. Rh/CoCe and Rh/Co exhibit essentially identical ∆*H* ^‡^ (ca. 63 kJ mol^−1^) and apparent ∆*S* ^‡^ values (ca. −135 J mol^−1^ K^−1^), indicating similar per‐site kinetics; the higher overall rate therefore arises from greater accessible site density and/or coverage effects related to the Rh–O–Co–O–Ce interfacial effects discussed earlier, rather than from a reduced enthalpic barrier. By contrast, Co and CoCe display comparable ∆*H* ^‡^ values (73 kJ mol^−1^) but measurably different apparent ∆*S* ^‡^ (−142 vs. −128 J mol^−1^ K^−1^). With the mechanism/RDS unchanged, the less negative apparent ∆*S* ^‡^ of CoCe indicates a less constrained transition‐state ensemble, consistent with the improved lattice oxygen activation, accounting for the activity difference of Figure 5a,b.

To gain insights into the mechanism of N_2_O decomposition, we evaluated the apparent reaction orders by examining the influence of the N_2_O partial pressure on the reaction rates under kinetic control (Figure 5d). Typically, N_2_O decomposition exhibits first‐order behavior in N_2_O when the RDS involves N_2_O dissociation. Deviations from first‐order kinetics are generally attributed to the influence of subsequent surface oxygen recombination [65, 95, 96]. Both Co and CoCe show apparent reaction order values of ca. 0.9, consistent with N_2_O dissociation as the RDS, with minimal influence from the subsequent oxygen evolution step. Therefore, as observed from the Arrhenius analysis, Ce addition does not alter significantly the fundamental reaction pathway but modulates the structural and electronic properties of Co_3_O_4_ by improving reducibility and weakening Co─O bonds. Conversely, Rh reduces the apparent reaction order to ca. 0.74 for both Rh/Co and Rh/CoCe, in agreement with Rh‐based catalysts [47, 61, 88]. The deviation from first‐order kinetics suggests a mechanistic shift in the nature of the active sites and/or the RDS. While Rh is known to facilitate N‐O dissociation, it can also promote surface–oxygen accumulation, hindering O_2_ desorption and rendering the recombination of surface oxygen species the new RDS. This variation highlights the critical role of Rh in altering the reaction pathway. Overall, the results are consistent with the Arrhenius parameters, confirming that Rh dictates the reaction pathway, whereas CeO_2_ primarily influences the structural and electronic environment of the active sites. In summary, Rh and Ce exist as highly dispersed, short‐range species anchored to the Co_3_O_4_ host, with Rh in an oxidized (RhO*_x_*) state. Both components modulate the oxidation state and local coordination of Co via defects and strong electronic interactions at the Rh–O–Co–O–Ce interface.

### 2.4 *Operando* X‐Ray Absorption Spectroscopy (XAS) and Reaction Mechanism

**FIGURE 6:**
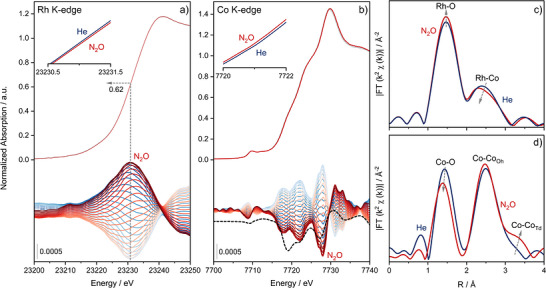
Modulated excitation experiments with alternate N_2_O/He and He cycles over Rh/CoCe. (a) Time‐resolved Rh K‐edge XANES spectra (top) and phase‐resolved spectra (bottom). (b) Time‐resolved Co K‐edge XANES spectra (top) and phase‐resolved spectra (bottom). The dashed line is the scaled difference between the spectra of Co_3_O_4_ and CoAl_2_O_4_ references. (c) Rh K‐edge phase‐uncorrected *k* ^2^‐weighted magnitude of the FT‐EXAFS spectra of Rh/CoCe in N_2_O/He and He environments. (d) Co K‐edge phase‐uncorrected *k* ^2^‐weighted FT‐EXAFS spectra in N_2_O/He and He environments. Conditions: 8000 ppm N_2_O, balance He, 1 atm, total flow rate of 100 mL min^−1^, catalyst mass of 25 mg.

Having established the structural and electronic properties of the most active catalyst in the series, we investigated the behavior of Rh/CoCe during N_2_O decomposition using *operando* modulated excitation XAS experiments (ME‐XAS). Alternated cycles of N_2_O/He (8000 ppm) and He mimic the redox nature of the N_2_O decomposition reaction, comprising an oxidation‐half‐cycle (OHC) and a reduction‐half‐cycle (RHC). Because the apparent N_2_O reaction order for Rh/CoCe indicates that Rh is responsible for N_2_O activation and decomposition, the interaction of N_2_O with the highly dispersed RhO*_x_* species was probed by Rh K‐edge XANES. The average time‐resolved Rh K‐edge XANES spectra (Figure 6a, top) show a subtle positive shift of the absorption edge under N_2_O‐rich conditions, consistent with oxidation of RhO*_x_* (RhO*_x_* → Rh*^n^* ^+1^O*_x_*_+1_) and a shift to lower energy during the He half‐cycle, indicating reduction of Rh*^n^* ^+1^O*_x_*_+1_ to RhO*_x_*. Phase‐sensitive detection (PSD; Figure 6a, bottom) enhances the sensitivity to these subtle changes providing unambiguous evidence of a reversible redox process (Figure S19), wherein N_2_O induces the oxidation of Rh (2N_2_O → 2N_2_ + 2O; OHC), which is marked by a positive feature at ca. 23231 eV, followed by the recombination/desorption of O_2_ that drives auto‐reduction of Rh (2O → O_2_; RHC) [47, 61, 88], in agreement with the rapid N_2_ evolution (*m*/*z* = 28) in the OHC and a slower O_2_ release (*m*/*z* = 32) in the RHC (Figures S20 and S21). This behavior further points toward the RHC as RDS. Similar results were obtained for Rh/Co_,_ albeit with lower PSD amplitude (Figure S22), validating the Rh‐mediated redox cycle consistent with the similar kinetic fingerprints of Rh/Co and Rh/CoCe (Figure 5c,d).

The intensity of the signal emphasized by PSD at 23231 eV scales linearly with N_2_O partial pressure (*p*_N2O_; *n*_N2O_ = 0.37; Figures S23 and S24), consistent with the measured reaction orders (*n*_N2O_ < 0.9) and the rate‐determining nature of the RHC, reflecting the inhibitory effect of surface O‐species accumulation at increasing *p*_N2O_ values [96]. Complete suppression of the PSD response in an identical ME‐PSD experiment conducted in excess of O_2_ in the feed (Figure S25) further verifies that, on Rh‐based catalysts, the RHC is dominated by inhibition of the Rh redox cycle due to oxygen build‐up, in line with the catalytic experiments (Figure S16b) [74, 97].

In identical ME‐XAS experiments at the Co K‐edge (Figure 6b, top), N_2_O induces a shift of the absorption edge toward lower energies, thus reduction of Co species, while in He the edge shifts to higher energies, indicating reoxidation. The PSD data clearly demonstrates the reversible nature of this process (Figure 6b, bottom) with spectral features closely resembling the spectral difference between Co_3_O_4_ and CoAl_2_O_4_. Therefore, Co species cycle between Co^3+^ (Co_Oh_) and Co^2+^ (Co_Td_) environments during N_2_O decomposition on Rh/CoCe.

Remarkably, the response at the Co K‐edge made visible by PSD is shifted by about 130° relative to that at the Rh K‐edge (Figure S26) providing direct *operando* evidence of a coupled Rh–O–Co redox mechanism, wherein RhO*_x_* and CoO*_x_* species undergo sequential oxidation–reduction events, beginning with oxidation of RhO*_x_* followed by reduction of CoO*_x_*. This cooperative mechanism is consistent with the interfacial effects inferred from the structural, electronic, and surface characterization results.

Variations in the local environment during N_2_O decomposition were probed by EXAFS. As shown in Figure 6c, the magnitude of the *k* ^2^‐weighted Rh K‐edge spectra of Rh/CoCe reveals that upon N_2_O introduction, the Rh–O contribution slightly increases, consistent with the oxidation of RhO*_x_* species in the N_2_O‐rich period and a higher Rh–O coordination number, likely arising from oxygen deposition following N─O bond cleavage. More pronounced structural changes were observed in the magnitude of the *k* ^2^‐weighted Co K‐edge spectra (Figure 6d). The amplitude of the Co–O contribution decreases upon contact with N_2_O, concomitant with an increase in the Co–Co_Td_ scattering contribution. The attenuation of the Co–O path agrees with the partial reduction of Co^3+^_Oh_ sites indicated by XANES, together with increased disorder and/or a lowered average coordination associated with defect formation. The gain in Co‐Co_Td_ is ascribed to lattice rearrangement upon formation of oxygen vacancies (V_O_) [60]. These results indicate that Co_3_O_4_ experiences dynamic and reversible structural changes that are complementary, though reciprocal, to those of Rh, underscoring their coupled redox behavior during N_2_O decomposition.

Based on our kinetic analysis and *operando* ME‐XAS, and supported by previous studies [47, 74, 88, 98, 99], we propose a cooperative RhO*_x_*/CoO*_x_* reaction mechanism for N_2_O decomposition over Rh/CoCe (Equations (1), (2), (3)). N_2_O decomposition initiates with the OHC via dissociative adsorption of N_2_O on RhO*_x_* (Rh^3+^/Rh^4+^), leading to N─O bond cleavage, N_2_ release, and oxidation of RhO*_x_* (**1**). The RHC proceeds via auto‐reduction of oxygen‐rich RhO*_x_* species (Rh*^n^* ^+1^O*_x_*_+1_) through a hetero‐recombination step, in which lattice oxygen from Co─O bonds in Co_3_O_4_ reacts with Rh‐bound oxygen to form O_2_, alongside reduction of Rh and creation of an oxygen vacancy (V_O_) (**2**). Finally, regeneration of the Co_3_O_4_ lattice through replenishment of V_O_ by N_2_O produces the second N_2_ molecule (**3**).

(1) $$\mathrm{Rh}-O_{x}+N_{2}O\to \mathrm{Rh}-O_{x+1}+N_{2}$$

(2) $$\mathrm{Rh}-O_{x+1\;}+{\mathrm{Co}}^{3+}-O_{\mathrm{latt}}\to \;\mathrm{Rh}-O_{x}+{\mathrm{Co}}^{2+}-V_{O}+O_{2}$$

(3) $$Co^{2+}-V_{O}+N_{2}O\;\to \;Co^{3+}-O_{\mathrm{latt}\;}+N_{2}$$

This cooperative Rh/Co sequence is consistent with a Mars‐van Krevelen mechanism, previously reported in the context of CO oxidation on Co_3_O_4_ and N_2_O decomposition on CuO‐Co_3_O_4_ catalysts [100].

To further clarify the role of CeO*_x_*, we used DFT to track charge redistribution along the key steps of the catalytic cycle on Rh/CoCe and, for comparison, on Rh/Co (Figure S27a,b). For both catalysts, Rh is oxidized upon N_2_O adsorption and cleavage (Equation 1) and reduced during O_2_ desorption (Equation 2), in agreement with the experimental evidence. However, the presence of CeO*_x_* significantly modifies the electronic structure along the reaction sequence. On the clean slab, Rh is more oxidized in Rh/CoCe (+1.26 |e|) than in RhCo (+1.20 |e|), leading to a more favorable N_2_O adsorption by 1.43 eV. Following N─O bond cleavage (Equation 1), N_2_ evolves and the remaining oxygen atom migrates to the surface forming a Rh–O–O–Co peroxide intermediate involving the Co^3+^_Oh_ sites of Co_3_O_4_ [68, 69, 70].

While this peroxide intermediate forms on both catalysts, it is more stabilized on Rh/CoCe, where the peroxo species binds to Rh through a single bond. Upon N_2_O activation, the Ce^3+^/Ce^4+^ redox couple undergoes partial reduction (average Ce charge changes from +2.28 |e| to +2.18 |e|), with Rh and Co mediating charge transfer from CeO*_x_* to the peroxo intermediate, resulting in more reduced oxygen species (average per O atom: −0.80 |e| and −0.37 |e| for Rh/CoCe and Rh/Co, respectively). Subsequently, after O_2_ desorption, CeO*_x_* moderates the reduction of interfacial Co^3+^_Oh_ sites, enabling them to retain a higher oxidation state compared to Rh/Co (+1.34 |e| vs. +1.16 |e|). This stabilization of both the initial and the final state lowers the thermodynamic cost of O_2_ release (Equation 2) by 0.71 eV compared to Rh/Co (Figure S27c). Taken together, these results show that, while Rh^3+^/Rh^4+^ sites drive N_2_O activation, CeO*_x_* promotes interfacial charge transfer via bridging oxygen atoms in the Co_3_O_4_ host, sustaining the redox cycle and enhancing catalytic activity.

## 3 Conclusion

The tunable structure and electronic properties of spinel metal oxides enable the design of a RhO*_x_*/Co_3_O_4_‐CeO_2_ catalyst with RhO*_x_* and CeO*_x_* species anchored and stabilized at short‐range on Co_3_O_4_. This engineered interface facilitates the formation of an expanded metal oxygen Rh–O–Co–O–Ce connectivity that enhances redox properties, promoting the weakening of the Co─O bonds and the activation of the lattice oxygen, delivering an efficient catalyst for the N_2_O decomposition. Distinct reaction pathways for Rh‐containing catalysts compared to Co_3_O_4_ and Co_3_O_4_‐CeO_2_ highlight the central role of Rh in the reaction mechanism in facilitating N‐O bond dissociation and modifying the rate‐determining step. *Operando* XAS measurements confirm a cooperative RhO*_x_*‐CoO*_x_* Mars‐van Krevelen mechanism, in which the Rh redox cycle is sustained by electron/oxygen exchange with the Co_3_O_4_‐(CeO*_x_*) matrix, underscoring the role of *operando* techniques enhanced by advanced experimental protocols for uncovering surface processes and revealing mechanistic details in complex catalysts. This work emphasizes the importance of engineered metal oxide–metal oxide interfacial interactions in the design of effective catalysts for industrially relevant redox reactions and provides a platform for the development of improved oxygen‐based catalysts.

## Author Contributions

**Daniel C. Cano‐Blanco**: conceptualization, investigation, writing – original draft, methodology, formal analysis, data curation, writing – review and editing. **Silvio Bellomi**: conceptualization, investigation, writing – original draft, methodology, formal analysis, writing – review and editing. **Daniele Bonavia**: formal analysis, investigation, writing – original draft, data curation, writing – review and editing. **Ivo Alxneit**: investigation, formal analysis, writing – review and editing. **Kirill A. Lomachenko**: investigation, writing – review and editing. **Karin Föttinger**: writing – review and editing, supervision, funding acquisition. **Oliver Kröcher**: writing – review and editing, supervision. **Davide Ferri**: supervision, project administration, writing – review and editing, funding acquisition, conceptualization.

## Conflicts of Interest

The authors declare no conflicts of interest.

## Supporting information



The authors have cited additional references within the Supporting Information [99, 101, 102, 103, 104, 105, 106, 107, 108, 109, 110, 111, 112, 113, 114, 115, 116, 117, 118, 119, 120, 121, 122, 123, 124, 125]. **Supporting File**: anie73090‐sup‐0001‐SuppMat.docx.

## Data Availability

The data that support the findings of this study are available from the corresponding author upon reasonable request.
