# Supplementary material for: Cooperative Redox Catalysis of N2O Decomposition by Short‐Range RhO x and CeO x Anchored to Co3O4
Source: Angew Chem Int Ed Engl. 2026 Jun 23;65(34):e7890563. doi: 10.1002/anie.7890563 (PMC13480521; doi:10.1002/anie.7890563)
Supplement: Supplementary file 1 [file ANIE-65-e7890563-s001.docx]

**Supporting Information**

**Cooperative Redox Catalysis of N_2_O Decomposition by Short-Range RhO_x_ and CeO_x_ Species Anchored to Co_3_O_4_**

Daniel C. Cano-Blanco,^[a],[b],‡^ Silvio Bellomi,^[c],‡^ Daniele Bonavia,^[a]^ Ivo Alxneit,^[a]^ Kirill A. Lomachenko,^[d]^ Karin Föttinger,^[c]^ Oliver Kröcher,^[a],[b]^ Davide Ferri^[a]^*

[a]	D.C. Cano-Blanco, Dr. D. Bonavia, Dr. I. Alxneit, Prof. O. Kröcher, Dr. D. Ferri
PSI Center for Energy and Environmental Sciences
Paul Scherrer Institute
CH-5232 Villigen PSI, Switzerland
E-mail: davide.ferri@psi.ch

[b]	D.C. Cano-Blanco, Prof. O. Kröcher
Institute of Chemical and Bioengineering
École polytechnique fédérale de Lausanne (EPFL)
CH-1015 Lausanne, Switzerland

[c]	Dr. S. Bellomi, Prof. K. Föttinger
Institute of Materials Chemistry
Technische Universität Wien
Getreidemarkt 9/165, 1060 Vienna, Austria

[d]	Dr. K. A. Lomachenko
European Synchrotron Radiation Facility
71 avenue des Martyrs, CS 40220, 38043 Grenoble Cedex 9, France

^‡^ These authors have contributed equally to this work

**Keywords:** Redox reactions, Metal oxide-metal oxide interface, Spinel, *operando*

**Table of Contents**

Experimental……………………………………………………………………………….S3

Supplementary Note 1: Modulated Excitation and Phase-Sensitive Detection……..S7

Supplementary Tables…………………………………………………………………….S8

Supplementary Figures…………………………...……………………………………..S17

Experimental

Catalyst Synthesis

*Co_3_O_4_*

Co_3_O_4_ (labelled as Co in the following) was synthesized by precipitation by the dropwise addition of a solution of sodium carbonate (Na_2_CO_3_; 1 M; Sigma-Aldrich) to an aqueous solution of Cobalt (II) nitrate hexahydrate (Co(NO_3_)_2_∙6H_2_O; Sigma-Aldrich). The resulting suspension was stirred for 3 h, filtered, and washed several times with distilled water (5 L). The solid was dried overnight at 110 °C (ca. 12 h) and calcined at 400 °C for 4 h to obtain the final Co_3_O_4_ powder.

*Co_3_O_4_-CeO_2_*

Co_3_O_4_-CeO_2_ (90 wt% Co, 10 wt% Ce; CoCe) was prepared by co-precipitation following a reported procedure ^[65]^. An aqueous solution of cerium (III) nitrate hexahydrate (Ce(NO_3_)_2_∙6H_2_O; 10 mL; Sigma-Aldrich) was added dropwise to a solution of Co(NO_3_)_2_∙6H_2_O (40 mL) under continuous stirring, followed by dropwise addition of Na_2_CO_3_ until pH = 9 to induce co-precipitation of the metal carbonates. The suspension was stirred for 3 h, filtered, washed with distilled water (5 L), dried overnight at 110 °C (ca. 12 h), and calcined at 400 °C to yield the final mixed oxide powder.

*RhO_x_/Co_3_O_4_ and RhO_x_/Co_3_O_4_-CeO_2_*

Rhodium (1 wt%) was incorporated by incipient wetness impregnation (IWI) using an aqueous solution of rhodium (III) nitrate (Rh(NO_3_)_3_; 10 wt% Rh; Sigma-Aldrich). The precursor solution volume was adjusted to coincide with the previously measured pore volume of each support and was added dropwise to Co_3_O_4_ and Co_3_O_4_-CeO_2_ under vigorous stirring, until forming a uniform paste without excess of liquid. The impregnated samples were dried overnight at 110 °C (ca. 12 h), ground to a fine powder, and calcined at 400 °C for 4 h, resulting in Rh/Co and Rh/CoCe samples.

Characterization

Elemental analysis was performed with inductively coupled plasma optical emission spectroscopy (ICP-OES).

Powder X-ray diffraction (PXRD) measurements were conducted with a D8 Advance diffractometer (Bruker) equipped with a Lynxeye XE detector and a 2.2 kW Cu anode long fine focus ceramic X-ray tube source (λ = 1.5406 Å). Data were recorded over a 2θ angular range of 5° to 70° with a step size of 0.02°.

Specific surface areas were determined by N_2_ physisorption at -196 °C using an Autosorb iQ (Quantachrome) instrument. Prior to the measurement, the samples were evacuated at 350 °C under vacuum overnight. The specific surface area was calculated using the Brunauer-Emmett-Teller (BET) method.

High energy X-ray total scattering data were collected at beamline ID15A of the European Synchrotron Radiation Facility (ESRF, Grenoble, France) at 90.48 keV. Pair distribution function (PDF) data were obtained using PDFgetX3 ^[101]^.

Raman spectra were obtained using a RAMANTouch microscope (Bruker) with a 532 nm excitation wavelength and 0.2 mW laser power.

High-angle annular darkfield (HAADF) scanning transmission electron microscopy (STEM) was performed using a probe-corrected JEM ARM-200 F (JEOL) microscope, equipped with a cold field emission gun (FEG) operated at 200 keV. HAADF-STEM images were collected at scattering angles of 68 < α < 250 mrad. Energy-dispersive X-ray (EDX) spectra were acquired using two silicon drift detectors (SDDs) with a combined solid-angle coverage of 1.7 sr.

X-ray absorption spectroscopy (XAS) experiments at the Co and Rh K-edges were performed at the BM23 beamline of the ESRF ^[102]^. Beam energy was calibrated using the corresponding metal foils prior to measurements. The storage ring was operating in uniform mode (200 mA current). The beam was monochromatized using a fixed-exit Si(111) double-crystal monochromator designed at ESRF. Higher harmonics at Co K-edge were removed by a pair of flat Si mirrors positioned at 3 mrad. At Rh K-edge, Pt-coated mirrors at 2.8 mrad were used. Measurements were conducted in transmission geometry using continuous scanning mode. Incident and transmitted X-rays were detected with gas-filled ionization chambers. Data processing for X-ray absorption near edge structure (XANES) and extended X-ray absorption fine structure (EXAFS) analysis was carried out using the Athena and Artemis software from the Demeter suite, and the Larch Python package ^[103, 104]^. Single- and multiple-scattering paths were calculated from the DFT-optimized structural models using FEFF.

Diffuse reflectance infrared Fourier transform spectroscopy (DRIFTS) of CO adsorption was performed on a spectrometer (Invenio, Bruker) equipped with a liquid nitrogen-cooled Mercury Cadmium Telluride (MCT) detector and a Praying Mantis mirror unit (Harrick). Spectra were collected at a resolution of 4 cm^-1^ with a scanner velocity of 80 kHz. Ca. 50 mg of catalyst was loaded in a custom-made spectroscopic cell fitted with a CaF_2_ window and a K-type thermocouple for temperature control [105]. CO adsorption was performed by exposing the sample to CO (1 vol% in Ar, Messer) under a total flow of 100 mL min^-1^ for 10 min, followed by purge with pure Ar flow for 10 min.

Hydrogen temperature-programmed reduction (H_2_-TPR) and oxygen temperature‑programmed desorption (O_2_-TPD) measurements were conducted using a ChemiSorb 2750 analyzer (Micromeritics) equipped with a thermal conductivity detector (TCD). The sample (ca. 100 mg) was loaded into a U-shaped quartz micro-reactor and tightened with quartz wool to form a packed bed. For H_2_-TPR, the sample was pretreated in O_2_ (10 vol% in Ar; Messer; 30 mL min^-1^) at 400 °C (heating rate; 10 °C min^-1^) for 1 h. After pretreatment, the reactor was cooled to 50 °C and purged with Ar. The sample was then exposed to H_2_ (5 vol% in Ar; Messer; 30 mL min^-1^) while heating to 750 °C (heating rate; 10 °C min^-1^). For O_2_-TPD, the sample was pretreated in He (30 mL min^-1^) at 400 °C (heating rate; 10 °C min^‑1^), cooled to 50 °C, and purged. It was then exposed to O_2_ (5 vol% in Ar; Messer; 30 mL min^-1^) for 1 h, followed by 30 min of He purge (30 mL min^-1^), and finally heated to 750 °C (heating rate; 10 °C min^-1^).

X-ray photoelectron spectroscopy (XPS) was conducted on a m-Focus 350 (SPECS) equipped with an Al K_α_ source and a PHOIBOS 150 WAL detector. The Al Kα source was operated at 7 mA and 14 kV. Sample charging was compensated using a flood gun operating at a voltage of 1 V and an emission current of 20 mA. The values were chosen as to match the observed and the theoretical value and expected shape of the O 1*s* signal. The data were calibrated using the main Co 2*p* signal. Scofield sensitivity factors and a Shirley background type were used for the fitting and quantification of the surface atomicity and speciation. Data analysis was performed using CasaXPS software.

Catalytic Activity and Kinetic Study

Catalytic performance was evaluated in a continuous plug-flow quartz reactor (i.d. = 4 mm) operated at atmospheric pressure. The sieved catalyst (100-150 µm; 50 mg) was diluted with sieved cordierite (100-150 µm; 50 mg) and packed between two quartz wool plugs. A K‑type thermocouple was inserted directly into the catalyst bed to monitor the reaction temperature. Gas flows were controlled using mass-flow controllers (Bronkhorst; EL-Flow series). Outlet gas concentrations were measured using an infrared spectrometer (Antaris IGS, Thermo Scientific) equipped with a heated gas cell and a mass spectrometer (Omnistar, Pfeiffer).

Prior to testing, the catalyst was heated to 450 °C (10 °C min^-1^) under Ar flow (100 mL min^‑1^) for 1 h. Activity was then evaluated under a total flow of 100 mL min^‑1^ of a model gas consisting of 500 ppmv of N_2_O in Ar, over the 75 – 550 ºC temperature range under conditions free of external mass transfer limitations. At each 25 °C increment, outlet concentrations were recorded after stabilization. The N_2_O conversion ($\text{χ}_{\text{N2O}}\text{)}$ was calculated as:

| $\text{χ}_{\text{N2O}}\text{= }\frac{{\text{[N}_{\text{2}}\text{O]}}_{\text{in}}\text{ – }{\text{[N}_{\text{2}}\text{O]}}_{\text{out}}}{{\text{[N}_{\text{2}}\text{O]}}_{\text{in}}}$ | **1** |
| --- | --- |

where [N_2_O]_in_ and [N_2_O]_out_, represent the inlet and outlet concentrations of N_2_O in ppmv, respectively.

Reactions rates were calculated according to:

| $\text{r}_{\text{ N2O}}\text{= }\frac{{\text{mmolN}_{\text{2}}\text{O}}_{\text{converted}}\text{ }}{\text{g}_{\text{cat }}\text{∙ h}}\text{ = }\frac{\text{F ∙ }{\text{[N}_{\text{2}}\text{O]}}_{\text{in}}\text{∙ }\text{χ}_{\text{N2O}}\text{ }\text{‧}\text{ 1000 ∙ 60}}{\text{m}_{\text{cat }}\text{∙ 22400 }}$ | **2** |
| --- | --- |

where [N_2_O]_in_ is the inlet N_2_O concentration in mole fraction, F is the total flow rate in mL min^‑1^, $\text{χ}_{\text{N2O}}$ the N_2_O conversion, m_cat_ the mass of catalyst in grams, and 22400 mL mol^-1^ is the molar volume at STP.

Apparent reaction orders were determined under differential conditions ($\text{χ}_{\text{N2O}}$ < 30%) at 375 °C. These conditions were achieved by varying the total flow (50-100 mL min^‑1^) and catalyst mass (1-30 mg), while maintaining a constant total bed mass of 100 mg trough dilution with cordierite. N_2_O concentration was varied between 100 and 700 ppmv. Reaction orders were obtained by plotting normalized reaction rates as a function of the corresponding partial pressure of N_2_O.

For wet N_2_O decomposition over Rh/CoCe, water (10 vol %) was added to the feed gas by reacting H_2_ and O_2_ in the desired concentrations on a Pt/Al_2_O_3_ catalyst at 180 °C. Stability tests were performed with Rh/CoCe in the same setup by feeding 100 mL min^‑1^ of a model gas consisting of 500 ppmv of N_2_O in Ar for 78 h.

**Modulated Excitation X-ray Absorption Spectroscopy**

*Operando* X-ray absorption spectroscopy (XAS) was carried out at beamline BM23 (ESRF) using the same setup described in the characterization section (*vide supra*). Continuous scanning was performed for both Rh and Co K-edges in transmission geometry, with a scan duration of 7 s and 11 s, respectively.

The reactor consisted of a custom-built stainless-steel cell ^[106]^ containing the catalyst packed between two quartz wool plugs to form a 1.0 x 0.5 cm (horizonal x vertical) bed with a depth of 1 mm. A thermocouple was inserted directly into the catalytic bed, enabling real-time temperature monitoring. The outlet of the cell was connected to a mass spectrometer (Omnistar GSD 320, Pfeiffer) for on-line gas analysis, while gas flows were controlled using mass-flow controllers (Bronkhorst; EL-Flow series).

For *operando* experiments under N_2_O decomposition conditions, the catalyst (10 - 30 mg) was diluted with cordierite and pretreated in He flow (100 mL min^-1^) by heating to 420 °C (heating rate; 10 °C min^‑1^), followed by a 30 min dwell time. The reactor was then cooled to the desired reaction temperature (400 °C). After temperature stabilization, data acquisition was initiated according to the following sequence: (i) 10 min in He, (ii) 10 min in N_2_O/He (2000 - 8000 ppmv, 10 min), followed by additional (iii) 10 min in He.

For the modulated excitation (ME)-XAS experiments, the sample underwent the same pretreatment procedure (420 °C; 10 °C min^-1^; 30 min dwell). The perturbation consisted of repeated periods of 4 min N_2_O/He (2000 - 8000 ppmv) alternated with 4 min He. A total of 10 periods were performed. Raw spectra were processed into phase-resolved datasets using a MATLAB script, as described in the **Supplementary Note 1**.

Computational Methods

Spin polarized DFT simulations were performed using the Vienna Ab Initio Simulation Package (VASP), version 6.3 ^[107, 108]^. The Perdew-Burke-Ernzerhof (PBE) generalized gradient approximation was employed with a plane-wave cutoff of 400 eV ^[109]^. The localization, strong on-site Coulomb repulsion, and proper description of Co 3*d* and Ce 4*f* orbitals were addressed using DFT + U, as implemented in the rotationally invariant scheme of Dudarev ^[110]^ with U-J values of 3.3 and 5.0 eV, respectively, and non-spherical contributions of the gradient of density in the PAW spheres ^[19, 111]^. The electronic convergence was eased using 0.05 eV of Gaussian smearing.

In agreement with the experimental conditions used, Co_3_O_4_ (110) was modeled using a slab composed of four 24 Co and 32 O centers stacked in the z-direction (eight atomic layers) and separated by a vacuum of 20 Å to avoid the interaction between periodic images ^[112, 113]^. The B‑type (110) surface of Co_3_O_4_ is dominant under the conditions used in this work ^[112, 113]^, and previously it was also effectively used to model materials for the N_2_O decomposition ^[48, 79]^; therefore, herein we only used such an oxygen-rich surface. Based on the obtained *ex-situ* characterization and in agreement with previous studies [111], the interface of CeO_x_ and the support was modeled using the global minimum configuration of a Ce_4_O_7_ cluster, presenting an average Ce oxidation state of 3.5 ^[114, 115]^. Guided by the extensive structural characterization performed, Rh was modeled as atomically dispersed atoms at the Co_3_O_4_ surface, substituting a Co octahedral site, and the relative position with respect to CeO_x_ chosen based on a systematic screening of different positions (**Fig. S1**).

To exclude spurious lateral interactions between the CeO_x_ clusters, a slab of size 11.3 × 16.1 Å was used. The geometry optimization was performed at Γ point only, using relaxation thresholds for electronic energies and ionic forces of 10^−5^ eV and 0.02 eV Å^-1^, respectively. The four bottom atomic layers of Co_3_O_4_ were kept frozen in the bulk configuration during the optimization, while the uppermost four layers, including CeO_x_ and atomically dispersed Rh, were allowed to relax. All the calculations included the long-range dispersion corrections as implemented in the DFT-D3 approach using zero-damping functions ^[116]^. The Co_3_O_4_ slab was chosen with an anti-ferromagnetic spin ordering along the z-axis, and the initial spin moments and signs were set according to the typical “checker-board” arrangement of bulk Co_3_O_4_ [117]. For all the simulations, the initial magnetic moments were set as ± 3.2 µB and 0 µB for Co ions in tetrahedral and octahedral positions, respectively. Rh and O were initialized using 0 µB, while all Ce atoms initial guesses were set at +1.0 µB.

Electronic structure analysis of the converged densities was performed using Γ-centered 8 × 6 × 1 Monkhorst-Pack grids ^[118]^. The center of an occupied electronic band (*i*) was calculated from the projected density of states (PDOS) using the following equation:

| $\text{ε}_{\text{i}}\text{=}\frac{\int_{\text{-∞}}^{\text{E}_{\text{F}}} \text{ε}\text{D}_{\text{i}}\text{(}\text{ε}\text{)d}\text{ε}}{\int_{\text{-∞}}^{\text{E}_{\text{F}}} \text{D}_{\text{i}}\text{(}\text{ε}\text{)d}\text{ε}}$ | **3** |
| --- | --- |

where $\text{εD}_{\text{i}}\text{(ε)}$ is the PDOS of the *i* orbitals weighted by the energy $\varepsilon$, and E_F_ is the Fermi energy ^[118]^. The same formula, but integrating the positive energy range was used to quantify the centroids of the unoccupied states.

The bulk structures for Co_3_O_4_ and the Rh-substituted octahedral and tetrahedral positions were optimized at the same theory level, but using an 8 × 8 × 8 Monkhorst-Pack grid. The structures are reported in **Fig. S.11**. The same setup was employed for the bulk references of Rh, Rh_2_O_3_ and RhO_2_ (**Fig. S2)** used to construct the calibration line correlating the Bader charges and the Rh oxidation states shown in **Fig. S15**. The accuracy of the oxidation states obtained using the calibration was quantified as 0.126 based on the uncertainty of the y-estimate of the linear regression.

The binding energies (ΔE) of intermediates were calculated using the optimized structures of the corresponding final and initial states. The Gibbs energies were evaluated using the ΔG = ΔE + ZPE − TΔS relation at 400 °C, where G, E, ZPE and TS are the variation of free energy, total energy, zero-point energy and entropic contributions, respectively. The entropy of solid-state surfaces was considered negligible.

Supplementary Note 1

Modulated Excitation and Phase-Sensitive Detection

Operando modulated-excitation (ME-XAS) experiments were conducted as described in the experimental section. The excitation was achieved by repeated equally long cycles of N_2_O/Ar and Ar flows while recording spectra at the Co and Rh K-edges.

Upon averaging the spectra over the modulation cycles the signal-to-noise ratio (S/N) is increased. The averaged spectra are then analyzed by lock-in amplification (phase-sensitive-detection, PSD). ME-XAS spectra were processed into new phase-resolved datasets using a Matlab software. For a sinusoidal stimulus, PSD is based on the following equation:

| $I\left( \phi^{PSD} \right)=\frac{2}{T}\int_{0}^{T} I\left( t \right)sin(k\omega t+\phi^{PSD})dt$ | **4** |
| --- | --- |

where ω (2π/T) is the stimulation frequency, k is the demodulation index (k = 1), and I(t) is the XAS intensity in the time-resolved spectra.

This treatment isolated the spectral response at the frequency of the applied stimulus (excitation), resulting in the phase-resolved spectra. The signal of the active species generally exhibits a time-delay relative to the stimulus, manifested in the phase-resolved spectra as a phase-delay ($\varphi$).

Supplementary Tables

**Table S1.** Summary of catalyst properties, including chemical composition from ICP-OES, BET specific surface areas and pore volumes.

| Catalyst | Chemical composition | | BET specific surface area / m^2^ g^-1^ | Pore volume / cm^3^ g^‑1^ |
| --- | --- | --- | --- | --- |
|  | **Ce**  **wt%** | **Rh**  **wt%** |  |  |
| Co | - | - | 66.3 | 0.323 |
| Rh/Co | - | 0.95 | 64.9 | 0.292 |
| CoCe | 8.9 | - | 133 | 0.589 |
| Rh/CoCe | 9.5 | 0.98 | 106 | 0.425 |

**Table S2.** Fitting parameters of the Co K-edge EXAFS spectra (S_0_^2^ = 0.736, calculated using a reference Co foil). N is the coordination number, R is the bond length, σ^2^ is the Debye-Waller factor, ΔE is the mismatch between theory and experiments, and the R-factor quantifies the goodness of fit.

| Sample | Path | N | R  / Å | σ^2^  / Å^2^ | ΔE  / eV | R factor |
| --- | --- | --- | --- | --- | --- | --- |
| Co | Co-O | 5.2 ± 0.7 | 1.905 ± 0.006 | 0.004 ± 0.001 | -8.7 ± 0.9 | 0.01 |
|  | Co-Co_Oh_ | 4.7 ± 0.5 | 2.852 ± 0.005 | 0.004 ± 0.005 |  |  |
|  | Co-Co_Td_ | 5.1 ± 0.6 | 3.357 ± 0.006 | 0.004 ± 0.005 |  |  |
| Rh/Co | Co-O | 5.4 ± 0.8 | 1.913 ± 0.005 | 0.003 ± 0.001 | -7.9 ± 0.9 | 0.01 |
|  | Co-Co_Oh_ | 5.1 ± 0.5 | 2.859 ± 0.005 | 0.004 ± 0.001 |  |  |
|  | Co-Co_Td_ | 5.9 ± 0.7 | 3.362 ± 0.006 | 0.004 ± 0.001 |  |  |
| CoCe | Co-O | 6.0 ± 0.8 | 1.909 ± 0.006 | 0.003 ± 0.001 | -8.4 ± 1.0 | 0.01 |
|  | Co-Co_Oh_ | 5.2 ± 0.6 | 2.856 ± 0.005 | 0.004 ± 0.001 |  |  |
|  | Co-Co_Td_ | 5.7 ± 0.7 | 3.359 ± 0.007 | 0.004 ± 0.001 |  |  |
| Rh/CoCe | Co-O | 5.8 ± 0.7 | 1.911 ± 0.005 | 0.003 ± 0.001 | -8.0 ± 0.9 | 0.009 |
|  | Co-Co_Oh_ | 5.3 ± 0.5 | 2.857 ± 0.005 | 0.005 ± 0.001 |  |  |
|  | Co-Co_Td_ | 5.8 ± 0.7 | 3.362 ± 0.006 | 0.004 ± 0.001 |  |  |

**Table S3.** Fitting parameters of the Rh K-edge EXAFS spectra (S_0_^2^ = 0.950). N is the coordination number, R is the bond length, σ^2^ is the Debye-Waller factor, ΔE is the mismatch between theory and experiments, and the R-factor quantifies the goodness of fit.^[a]^

| Sample | Path | N | R  / Å | σ^2^  / Å^2^ | ΔE  / eV | R factor |
| --- | --- | --- | --- | --- | --- | --- |
| Rh/Co | Rh-O | 5.38 ± 0.4 | 2.014 ± 0.006 | 0.003 ± 0.0009 | -5.9 ± 1.6 | 0.04 |
|  | Rh-Co | 3.32 ± 0.4 | 2.906 ± 0.009 | 0.006 ± 0.0018 |  |  |
| Rh/CoCe | Rh-O | 5.38 ± 0.5 | 2.018 ± 0.006 | 0.003 ± 0.0008 | -5. 6 ± 1.5 | 0.04 |
|  | Rh-Co | 3.08 ± 1.03 | 2.920 ± 0.011 | 0.009 ± 0.0024 |  |  |

^[a]^ Single- and multiple-scattering paths were calculated with FEFF from CIF files of the DFT-optimized structural models

**Table S4.** Summary of Co and Rh surface compositions as well as Co^2+^/Co^3+^ and Rh^3+^/Rh^4+^ ratios, as determined from XPS.

| Catalyst | Co^3+^  / eV | Co^2+^  / eV | Co^2+^/Co^3+^  / - | Rh^3+^  / eV | Rh^4+^  / eV | Rh^3+^/Rh^4+^  / - |
| --- | --- | --- | --- | --- | --- | --- |
| Co | 779.20 | 780.66 | 1.10 | - | - | - |
| Rh/Co | 779.20 | 780.65 | 1.00 | 309.15 | 310.15 | 9.59 |
| CoCe | 779.20 | 780.54 | 0.81 | - | - | - |
| Rh/CoCe | 779.20 | 780.67 | 0.75 | 309.25 | 310.05 | 1.37 |

**Table S5.** Summary of the XPS fitting for the O 1*s* region, as determined from XPS.

| Catalyst | O_I_  / eV | O_II_  / eV | O_III_  / eV |
| --- | --- | --- | --- |
| Co | 529.41 | 530.81 | 533.06 |
| Rh/Co | 529.30 | 530.64 | 532.50 |
| CoCe | 529.33 | 530.64 | 533.27 |
| Rh/CoCe | 529.18 | 530.39 | 532.30 |

**Table S6.** Summary of Ce surface composition and Ce^3+^/(Ce^3+^+Ce^4+^) ratios, as determined from XPS analysis.

|  |  | Ce^4+^  / eV | Ce^3+^_1  / eV | Ce^3+^  / eV | Ce^3+^_2  / eV | Ce^3+^_1  / eV | $\frac{\text{Ce}^{\boldsymbol{3+}}}{\text{Ce}^{\boldsymbol{3+}}\boldsymbol{+}\text{Ce}^{\boldsymbol{4+}}}$  / - |
| --- | --- | --- | --- | --- | --- | --- | --- |
| CoCe | **Position** | 883.81 | 889.73 | 886.50 | 898.35 | 882.00 | - |
|  | **%At** | 13.62 | 12.58 | 11.63 | 12.45 | 9.73 | 0.36 |
| Rh/CoCe | **Position** | 883.5 | 889.84 | 886.50 | 898.17 | 881.97 | - |
|  | **%At** | 12.67 | 11.23 | 12.65 | 12.15 | 11.30 | 0.40 |

**Table S7.** Summary of catalytic activity measurements.

| Catalyst | T_50_ ^[a]^  / °C | T_90_ ^[b]^  / °C | r_N2O_ ^[c]^  / mmolN_2_O g^-1^ h^-1^ | E_a, app_ ^[d]^  / kJ mol^‑1^ | lnA_app_ ^[e]^  / - | n_N2O_ ^[f]^  / - |
| --- | --- | --- | --- | --- | --- | --- |
| Co | 412.4 | 512.2 | 0.67 | 78.2 | 14.1 | 0.92 |
| Rh/Co | 312.1 | 399.5 | 3.01 | 69.5 | 14.9 | 0.72 |
| CoCe | 339.1 | 409.5 | 2.38 | 78.1 | 15.6 | 0.91 |
| Rh/CoCe | 250.4 | 292.5 | 12.96 | 64.1 | 14.8 | 0.74 |

^[a]^ Temperature of 50% N_2_O conversion

^[b]^ Temperature of 90% N_2_O conversion

^[c]^ Reaction rate, calculated at 375 °C using an inlet concentration of 500 ppmv N_2_O in an Ar carrier gas, under differential reaction conditions ($\text{χ}_{\text{N2O}}$ < 30%)

^[d]^ Apparent activation energy

^[e]^ Apparent pre-exponential factor

^[f]^ Apparent reaction orders

**Table S8.** Overview of reaction rates for N_2_O decomposition over various catalysts.^a^

|  | Rh wt% | r_N2O_  / mmolN_2_O g^-1^ h^-1^ | Conditions | Reference |
| --- | --- | --- | --- | --- |
| Rh/CoCe | 1 | 12.98 | 375 °C, 500 ppm N_2_O, F = 100 mL min^-1^, m_cat_ = 100 mg | This study |
| Rh/Co | 1 | 3.09 |  |  |
| CoCe | - | 2.42 |  |  |
| Co | - | 0.67 |  |  |
| RhO_x_/ZrO_2_ | 5 | 48.0 | 350 °C, 1000 ppm N_2_O, F = 100 mL min^-1^, m_cat_ = 170 mg | [47] |
| RhO_x_/TiO_2_ | 5 | 18.7 |  |  |
| RhO_x_/CeO_2_ | 5 | 41.2 |  |  |
| RhO_x_/Al_2_O_3_ | 5 | 28.1 |  |  |
| Co_3_O_4_ | - | 0.46 | 300 °C, 2000 ppm N_2_O, F = 50 mL min^-1^, m_cat_ = 200 mg | [119] |
| Co_3_O_4_ | - | 0.23 | 210 °C, 1000 ppm N_2_O, F = 150 mL min^-1^, m_cat_ = 500 mg | [65] |
| CoCe_0.05_ | - | 0.37 |  |  |
| Co_3_O_4_-CeO_2_ | - | 0.35 | 360 °C, 1000 ppm N_2_O, F = 50 mL min^-1^, m_cat_ = 300 mg | [120] |

^[a]^ Literature data, as published, has been re-calculated in terms of mass-intrinsic activity.

**Table S9.** Summary of apparent activation energies (E_a,app_) determined for N₂O decomposition on different catalytic systems.

|  | Rh wt% | E_a,app_ | Reference |
| --- | --- | --- | --- |
| Rh/CoCe | 1 | 64.1 kJ mol^-1^ | This study |
| Rh/Co | 1 | 69.5 kJ mol^-1^ |  |
| CoCe | - | 78.1 kJ mol^-1^ |  |
| Co | - | 78.2 kJ mol^-1^ |  |
| RhO_x_/ZrO_2_ | 1 | 132 kJ mol^-1^ | [47] |
| RhO_x_/ZrO_2_ | 5 | 111 kJ mol^-1^ |  |
| RhO_x_/CeO_2_ | 1.9 | 59.9 kJ mol^-1^ | [99] |
| Rh/Mg/Al | 1.8 | 103 kJ mol^-1^ | [123] |
| Co_3_O_4_ | - | 89.6 kJ mol^-1^ | [31] |
| Co_3_O_4_ | - | 63 kJ mol^-1^ | [121] |
| Zn_0.4_Co_2.6_O_4_ | - | 61 kJ mol^-1^ | [121] |
| Co_3_O_4_ | - | 74 kJ mol^-1^ | [119] |
| Cu_0.15_CoO_y_ | - | 66.3 kJ mol^-1^ | [122] |
| Co_3_O_4_-CeO_2_ | - | 84.3 kJ mol^-1^ | [120] |

Supplementary Figures

**Figure S1.** Different Co sites (blue) evaluated at the CeO_2_-Co_3_O_4_ interface (Ce: yellow, O: red) for substitutional Rh, and corresponding energy difference with respect to the most stable (position 4). Values are in eV. The most stable position is enclosed in a green dashed box.

**Figure S2.** Optimized bulk structure for the reference Rh structures: (a) metallic Rh, (b) Rh_2_O_3_ and (c) RhO_2_.

**Figure S3.** N_2_ physisorption isotherms. Data are offset for clarity.

**Figure S4.** Pair distribution functions (PDFs) of Co, CoCe, Rh/Co, and Rh/CoCe.

**Figure S5.** EDX sum spectrum of Rh/CoCe corresponding to the elemental maps of Fig. 1.

**Figure S6.** DRIFTS spectra of CO adsorption on Rh/CoCe and Rh/Co. Data are offset for clarity. Experimental conditions: 50 °C, 1000 ppm CO/Ar, 100 mL min^-1^.

The diffuse reflectance infrared (DRIFT) spectra of Rh/CoCe and Rh/Co during exposure to CO display a prominent signal at 2098 cm^-1^ of CO adsorbed on oxidized Rh that is accompanied by a shoulder at ca. 2110 cm^-1^ and a signal at 2020 cm^-1^, corresponding to the vibrational modes of Rh(CO)_2_ species characteristic of CO adsorption on atomically dispersed Rh^+ [124, 125]^. The short-range nature of Rh species anchored on Co_3_O_4_ is confirmed by the absence of features due to metal Rh aggregates, namely the linear CO band at ca. 2068 cm^-1^ and the bridged CO band at 1950-1850 cm^-1^.

**Figure S7.** *k*^3^-weighted Fourier-transformed Co K-edge EXAFS signals. The magnitude and real part of the spectra are vertically offset for clarity. The *k*^3^ weighting, *k*-range of 3 ~ 13 Å^−1^ and R range of 1.0~ 3.5 Å were used for the fitting of the Co K-edges. Single- and multiple-scattering paths were calculated from the DFT-optimized structural models using FEFF.

**Figure S8.** *k*^3^-weighted k-space Co K-edge EXAFS spectra and corresponding fitting curves. The *k*^3^ weighting, *k*-range of 3 ~ 13 Å^−1^ and R range of 1.0~ 3.5 Å were used for the fitting of the Co K-edges. Single- and multiple-scattering paths were calculated from the DFT-optimized structural models using FEFF.

**Figure S9.** *k*^3^-weighted Fourier-transformed Rh K-edge EXAFS signals of Rh/Co and Rh/CoCe samples. (a) R-space spectra showing, for each material, the magnitude and real part, which are plotted together and vertically offset for clarity. The *k*^3^ weighting, *k*-range of 2.6 ~ 12 Å^−1^ and R range of 0.5 ~ 6 Å were used for the fitting of the Rh K-edges. Single- and multiple-scattering paths were calculated from the DFT-optimized structural models using FEFF.

**Figure S10.** *k*^3^-weighted k-space Rh K-edge EXAFS spectra and corresponding fitting curves for Rh/Co and Rh/CoCe samples. The *k*^3^ weighting, *k*-range of 2.6 ~ 12 Å^−1^ and R range of 0.5 ~ 6 Å were used for the fitting of the Rh K-edges. Single- and multiple-scattering paths were calculated from the DFT-optimized structural models using FEFF.

**Figure S11.** DFT simulation of the Rh substitution into bulk Co_3_O_4_: a) thermodynamic stability and c) Rh oxidation state.

**Figure S12.** a) O_2_-TPD profiles of Co, CoCe, Rh/Co, and Rh/CoCe. b) Integrated desorption areas obtained after baseline correction using an asymmetric least-squares (ALS) method.

**Figure S13.** Co *2p* XPS spectra of Co, CoCe, Rh/Co, and Rh/CoCe.

**Figure S14.** Ce 3*d* XPS spectra of Rh/Co and Rh/CoCe.

**Figure S15.** Relation between the Bader charge of Rh (DFT) from known references and its oxidation state in Rh/Co and Rh/CoCe.

**Figure S16.** a) N_2_O decomposition under wet conditions over Rh/CoCe. Experimental conditions: 500 ppm N_2_O, N_2_ balance, 10 vol% H_2_O, and a flow rate of 100 mL min^-1^. b) Effect of oxygen on N_2_O decomposition over Rh/CoCe. Experimental conditions: 500 ppm N_2_O, N_2_ balance, 0 vol% H_2_O, 0-10 vol% O_2_, 250 °C, and a flow rate of 100 mL min^-1^.

**Figure S17.** Stability test during N_2_O decomposition over Rh/CoCe at 400 °C. Experimental conditions: 500 ppm N_2_O, N_2_ balance, 0 vol% H_2_O, 0 vol% O_2_, 400 °C, and a flow rate of 100 mL min^-1^.

**Figure S18.** Eyring-Polanyi plots for N_2_O decomposition constructed from light-off data. Solid lines are linear fits whose slopes give -∆H^‡^/R and intercepts yield ln (k_B_/h) + ∆S^‡^/R. Rates are normalized per gram of catalyst under fixed partial pressures; hence ∆S^‡^ values are apparent on this basis. ∆H‡ values units are kJ mol^-1^ and ∆S‡ units are J mol^-1^ K^-1^.

**Figure S19.** Temporal evolution of the 23232 eV signal recorded during the Rh K-edge modulated excitation (ME) experiment with alternating N_2_O/He and He cycles over Rh/CoCe catalyst.

**Figure S20.** Three ionization fragments measured during the Rh K-edge modulated excitation (ME) experiment with alternating N_2_O/He and He cycles over Rh/CoCe catalyst. 5 periods are shown.

**Figure S21.** Three ionization fragments measured during an oxidation half-cycle and a reduction half-cycle of a modulated excitation (ME) experiment with alternating N_2_O/He and He pulses over Rh/CoCe at the Rh K-edge.

**Figure S22.** Modulated excitation experiments with periodic N_2_O/He and He cycles over Rh/Co. Time-resolved Rh K-edge XANES spectra (top) and phase-resolved spectra (bottom). Experimental conditions: 400 °C, 8000 ppm N_2_O/He, He balance, flow rate of 60 mL min^-1^. Spectra are displayed within ϕ^PSD^ = 0-180° in 10° increments.

**Figure S23.** Phase-resolved Rh K-edge spectra of Rh/CoCe obtained during the modulated-excitation experiments at different N_2_O/He concentrations (2000, 5000, and 8000 ppm). Experimental conditions: 400 °C, 2000-8000 ppm N_2_O/He, He balance, flow rate of 60 mL min^-1^. Spectra are displayed within ϕ^PSD^ = 0-180° in 10° increments.

**Figure S24.** Correlation between the absolute maximum amplitude extracted from phase-resolved spectra of Rh/CoCe at the Rh K-edge during modulated excitation experiments (**Fig. S23**) and the N_2_O partial pressure (p_N2O_).

**Figure S25.** Modulated excitation (ME) experiments performed over Rh/CoCe under periodic N_2_O+O_2_/He and O_2_/He cycles. Time-resolved Rh K-edge XANES spectra (top) and phase-resolved spectra (bottom). Experimental conditions: 400 °C, 8000 ppm N_2_O/He, O_2_ 2% vol, He balance, flow rate of 60 mL min^-1^. Spectra are displayed within ϕ^PSD^ = 0-180° in 10° increments.

**Figure S26.** Phase-angle evolution of the 23232 eV (Rh K-edge) and 7728 (Co K-edge) signal recorded during the Rh K-edge and Co K-edge modulated excitation (ME) experiment with alternating N_2_O/He and He cycles over Rh/CoCe catalyst.

**Figure S27.** DFT simulations of the effect of CeO_x_: a) optimized structures of the main catalytic steps, b) Bader charge (q) of the components of the catalytic center, c) N_2_O adsorption and O_2_ desorption Gibbs free energies calculated at 673 K.
